# Multi-structural molecular docking (MOD) combined with molecular dynamics reveal the structural requirements of designing broad-spectrum inhibitors of SARS-CoV-2 entry to host cells

**DOI:** 10.1038/s41598-023-42015-2

**Published:** 2023-09-29

**Authors:** Anqi Da, Meritxell Wu-Lu, Jovan Dragelj, Maria Andrea Mroginski, Kourosh H. Ebrahimi

**Affiliations:** 1 Institute of Pharmaceutical Science, King’s College London, London, UK; 2 Institute of Chemistry, Technische Universität Berlin, Berlin, Germany

**Keywords:** Computational biology and bioinformatics, Protein analysis, Virtual drug screening, Drug discovery, Biologics

## Abstract

New variants of SARS-CoV-2 that can escape immune response continue to emerge. Consequently, there is an urgent demand to design small molecule therapeutics inhibiting viral entry to host cells to reduce infectivity rate. Despite numerous in silico and in situ studies, the structural requirement of designing viral-entry inhibitors effective against multiple variants of SARS-CoV-2 has yet to be described. Here we systematically screened the binding of various natural products (NPs) to six different SARS-CoV-2 receptor-binding domain (RBD) structures. We demonstrate that Multi-structural Molecular Docking (MOD) combined with molecular dynamics calculations allowed us to predict a vulnerable site of RBD and the structural requirement of ligands binding to this vulnerable site. We expect that our findings lay the foundation for in silico screening and identification of lead molecules to guide drug discovery into designing new broad-spectrum lead molecules to counter the threat of future variants of SARS-CoV-2.

## Introduction

Severe acute respiratory syndrome coronavirus 2 (SARS-CoV-2) first appeared in December 2019 in Wuhan, Hubei province China^1^. Until June 2022, there have been more than 550 million confirmed covid-19 infections worldwide, and the number of deaths is reportedly over six million individuals (WHO Coronavirus Statistics 2022). The global pandemic of the virus and the lack of therapeutics to counter severe disease put high pressure on the medical care system in many countries and have had substantial social, religious, economic, financial, and political burdens. An unprecedented joint effort of scientists around the world led to the development of vaccines and therapies inhibiting viral replication machinery, e.g. remdesivir, to protect the most vulnerable population and reduce mortality rates.

**Figure 1:**
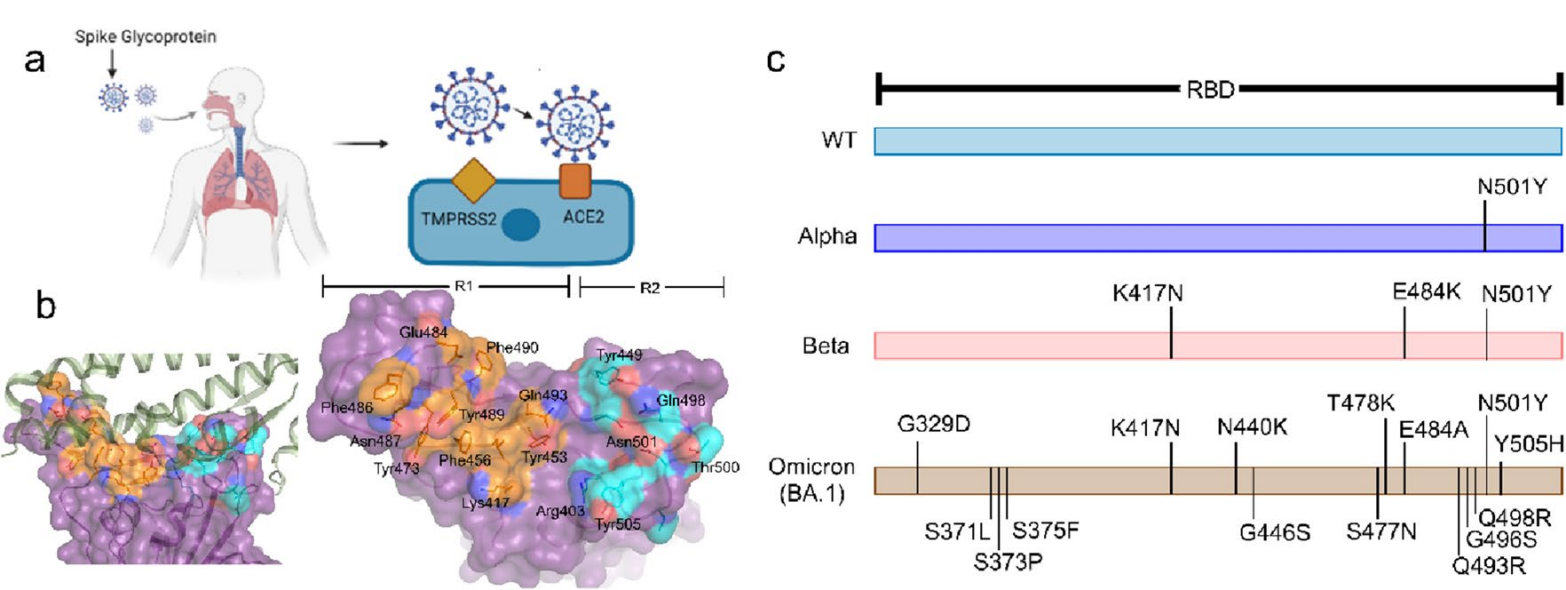
SARS-CoV-2 and its entry process. (**a**) SARS-CoV-2 entry into nasal epithelial cells is a complex process that requires the proteolytic processing by TMPRSS2 to induce conformational changes on spike protein and concerted action of RBD binding to ACE2 receptor. (**b**) Structure of RBD in complex with ACE2 receptor (green) (PDB code: 7C8D). The two regions of RBD encompassing the key amino acid residues interacting with the ACE2 receptor are coloured orange (R1) and cyan (R2). (**c**) Mutations of the RBD of three major variants are shown as compared to wild-type (WT) RBD.

It is now clear that SARS-CoV-2 infects host cells using its homotrimeric surface spike glycoprotein (S protein)^2,3^. This protein comprises two functional subunits: S1 subunit, responsible for the attachment of the virus to the host cell receptor, and S2 subunit, responsible for the fusion of the viral and cellular membranes^4^. The S protein is cleaved at the boundary of S1 and S2, remaining non-covalently bound in the prefusion conformation, then further cleaved by transmembrane serine protease 2 (TMPRSS2) inducing irreversible conformational changes for binding to the cellular receptor angiotensin-converting enzyme 2 (ACE2) (Fig. 1a). The distal domain of S1, also known as the receptor-binding domain (RBD) (Fig. 1b), is responsible for ACE2 recognition, similar to the mechanism described for SARS-CoV-1^5^. The pocket of RBD interacting with the ACE2 receptor can be divided into R1 and R2 pockets (Fig. 1b) based on the mutations occurring on different major variants affecting RBD-binding to the ACE2 receptor (Fig. 1c). Mutations of the S protein, particularly those in the R1 and R2 pocket of RBD (Fig. 1c), can lead to the rise of variants scaping antibody response and increased RBD binding affinity to the ACE2, undermining the efficacy of current therapies and vaccines^6–9^. These mutations have led to the rise of new variants, namely Alpha, Beta or Omicron (BA.1) (Fig. 1c). The mutational landscape of the RBD is predicted to lead to new variants of concern with the ability to scape antibody response^9^. Hence, there is a growing interest in discovering broad-spectrum antiviral small molecules binding to R1, R2, or the R1–R2 interface and interfering with the viral entry process to counter the emergence of future variants of concern.

In silico screening, approaches have become an integral part of drug discovery pipelines to enable identification of the best leads in initial stages and reduce costs^10–13^. Computational studies have largely been used to identify lead natural products (NPs) and other small molecules and nanobodies interfering with RBD binding to the ACE2 receptor^12,14–23^. Multiple conformation-based virtual screening (ensemble docking) have been introduced to identify SARS-CoV-2 main protease (M^pro^) inhibitors among commercially available compounds^24^. Some of these predictions are confirmed using biochemical studies suggesting that in silico studies using modern computational methods are suitable for discovering lead molecules and guiding drug discovery^19,24–26^. Computational studies take advantage of the structure of RBD solved in the presence of a binding partner, i.e. ACE2, antibody, or nanobody. In these studies, the protein partner is removed for docking studies. Most computational studies have not tested how certain conformational changes induced in the RBD structure by the binding of protein-partner (ACE2, antibody, or nanobody) can affect in silico screening. Additionally, a vulnerable pocket to which a small molecule ligand can bind, irrespective of the RBD mutations, has yet to be reported. Such a vulnerable pocket is comparable to the vulnerable epitopes of broad-spectrum neutralization antibodies^27–31^.

Here, we aimed to identify new natural products with the ability to interfere with RBD-binding to the ACE2 receptor. We used in silico multi-structural molecular docking (MOD) screening combined with molecular dynamics (MD) calculations. We systematically screened the binding of more than 70 NPs using three structures of wild-type (WT) RBD solved in the presence of ACE2, antibody, or nanobody and those of three major variants: alpha, beta, and omicron. We tested some of the already identified compounds (SI Table 1) to check if their bindings are affected by the structure of RBD. The results revealed a vulnerable pocket of RBD and the structural details of lead ligands targeting this pocket. The findings suggest key structural requirements for discovering and synthesizing new ligands as broad-spectrum inhibitors of SARS-CoV-2 entry to host cells.

## Methods

### Molecular docking

Molecular docking simulations were performed using PyRx open-source software, taking advantages of Auto Dock Vina program^32^. The specific operation method, including preparing input files for docking and running virtual screening using vina wizards are described step by step in Chemical Biology (Dalla Kyan S, Olson A, 2014)^33^. It is worth noting that the ACE2, antibody and nanobody fragments in the RBD structures were removed by modifying the protein PBD text file to make the structures suitable for molecular docking. The water molecules in RBD structures were removed when docking since the structures were solved in the absence of a small molecule ligand and the binding pocket of a small molecule ligand could be occupied with water molecules. The Vina search space was performed to cover the top domain of RBD where it interacts with the ACE2 receptor (SI Fig. 1a). An example of the Vina search space defined for molecular docking is shown below^11^ (PDB Code: 7F63). The Vina search space parameters were:

$${\text{Center}}:{\text{ X}}:{ 175}.{4453};{\text{ Y}}:{ 199}.{3489};{\text{ Z}}:{ 159}.{6444}$$

$${\text{Dimensions }}\;\left( {{\text{{\AA}ngstrom}}} \right):{\text{ X}}:{\text{ 41}}.{\text{8118}};{\text{ Y}}:{\text{ 5}}0.{\text{7784}};{\text{ Z}}:{\text{ 34}}.00{\text{94}}$$

After virtual screening is completed, PyRx automatically advances to Analyse Results page, where results of virtual screening computation can be viewed. Auto Dock Vina, by default, outputs the nine best binding modes for each ligand and mode with highest binding energy for each ligand was selected for further analysis. It should be emphasized that we ignore unreasonable binding models in which the ligand binds to the side or back of the top region of RBD (SI Fig. 1b), lacking the interaction with the ACE2 receptor.

### Calculation of dissociation constant (*K*_*d*_)

Th protein–ligand-solvent mixture is a complex thermodynamic system containing complicated interactions and heat exchange. The driving forces that determine the association between RBD and ligands are a combination of various interactions and energy exchanges among the RBD, ligands and solvent. Gibbs free energy, known as a thermodynamic potential that could be used to measure the maximum amount of work in a reversible progress performed by a thermodynamically closed system at constant temperature and pressure. As with all spontaneous processes, Gibbs free energy is minimized when a system reaches chemical equilibrium at constant pressure and temperature, the decrease of which equals to the work done by the system to its surroundings, minus the work of pressure forces. The accuracy of the PyRx for predicting the binding of a molecule was tested in the previous study^34^. We estimated an error of ± 0.55 (kCal/mol) on the Gibbs free energy getting from PyRx and use a ΔG value of -8.3 ± 0.55 (kCal/mol)^34^ as a benchmark for identifying NPs with reasonable binding affinity to RBD. ΔG is used to calculate the dissociation constant (*K*_*d*_) (µM) using formula: K_d_ = c^Ө^ × e $\left( {\frac{{{\Delta G}}}{R \times T}} \right)$, where c^θ^ is the reference concentration (1 mol/l), R is the universal gas constant (1.98 cal/K mol) and *T* is the temperature in degrees of Kelvin (310 K). The equilibrium dissociation constant (*K*_*d*_) is the basic parameter to evaluate the binding properties of the drug-receptor, which presents the extent of ligand leaving the protein. Hence, ligands having higher binding affinity to RBD should have smaller *K*_*d*_.

### Molecular modelling and dynamics

In this study we have set up 6 different systems corresponding to two ligands identified using MOD, i.e. *ligand viii* and *ligand ix,* complexed with glycosylated RBD of wild-type (PDB Code: 7C8D, 7F63 and 7KGJ) or mutated structures: Alpha (PDB Code: 7NEG), Beta (PDB Code: 7PS0) and Omicron (PDB code: 7WBP). The initial coordinates of the protein and ligand were taken after docking the ligand to the RBD. Initial modelling of RBDs was performed using CHARMM^31^ software and CHARMM 36 Force Field^32^. The protonation pattern of the protein (RBD) was determined Karlsberg2+^35^, assuming a pH 7.0. Hence, all titratable residues were in their standard protonation state and His519 and His505 in the neutrally charged state (δ-tautomer).

The preparation of the ligand was first optimized quantum chemically using Gaussian16 as we described previously^36^. The force field parameters of the ligand were generated with Ligand Modeler tool of CHARM-GUI Web Interface^37^. The atomic partial charges of the ligands of interest were calculated from B3LYP/6-31G** optimized wave functions, using the electrostatic potential-fitted charge calculation protocol (ESP charges) using JAGUAR^38^. To complete the molecular dynamics (MD) simulation setups, the structures of ligand and RBD complexes were neutralized with ions and solvated in a TIP3P water box with periodic boundary conditions (box size: 15 Å × 15 Å 15 Å) as shown in SI Fig. 2. All MD simulations were performed using the same conditions with NAMDV2.14 software, with 2 fs time step using Shake algorithm to fix bond lengths of hydrogen atoms and Langevin dynamics at 300 K and flexible cell size. The resulting structures were then energy minimized and thermally equilibrated with harmonic constrains applied to all heavy atoms and sugar dihedrals angles. To ensure stability of isolated RBD constraints were applied in the production run as follows: sugar and heavy atoms retained harmonic constraints from equilibration, except for the selected residues present on the binding pockets of RBD in both WT and mutated structures within 3 Å as in our previous work^39^. All MD simulations were repeated 3 times and were each 100 ns long. The thermal stability of the resulting structures was evaluated by computing the root-mean-square-deviations of heavy atoms (RMSD) with respect to initial structural models, the root-mean-square-fluctuations (RMSF) of heavy atoms of the ligands and their binding pocket, the radius of gyration (RG) of the protein–ligand complex and the solvent-accessible-surface-area (SASA) of the ligand and the protein. All these quantities were calculated using plugins of Visual Molecular Dynamics (VMD)^57^. In addition, interaction energies between ligands and binding pocket were computed using INTER subroutine of CHARMM^31^ software using structures from time frames extracted every 100 ps from each MD trajectory. For this purpose, we have considered the electrostatic (Coulomb) and the Van der Waals components of the total interaction energies. VMD code was also employ for visualization of structures and generation of images.

## Results and discussion

### In silico screening predicts multiple NPs interfering with RBD-binding to the ACE2

Previous biochemical and in silico studies have revealed that some NPs, including bile acids like tauro-α-muricholic acid^20,25^, β-amyrin^40^, folic acid^41^, vitamin B6^42^, campesterol and stigmasterol^43^ can bind to R1, R2, or at the interface of R1–R2 interfering with the interactions between the RBD and the ACE2 receptor. These natural products thus, are proposed to have therapeutic benefits. We aimed to expand previous studies and screen the binding of 70 NPs to the RBD using in silico molecular docking and identify those interfering with the RBD-binding to the ACE2 receptor. To perform docking studies, we first chose the structure of a WT-RBD solved in the presence of ACE2 receptor. To screen for the ligands that can bind to the RBD surface interacting with the ACE2 receptor (Figs. 1b and S1), we removed the ACE2 from the structure and defined the RBD-ACE2 interface including R1 and R2 pocket (Fig. 1b) as the docking pocket (Methods). We used PyRX open source software^33^, which can predict the binding site of ligands accurately^20,33^, and screened the binding of NPs from various sources (Supplementary Methods) (SI Table 1). The dissociation constant was calculated from the Gibbs free energy ΔG given by PyRx (Methods). Among all NPs tested, eight ligands have ΔG values close to the previously reported benchmarked value of − 8.3 ± 0.55 (kCal/mol)^20^ (Table 1). These ligands have some structural similarities: they all have aromatic and aromatic hydroxyl groups. They interact with the RBD via hydrogen bonds, dipole–dipole interactions, and hydrophobic interactions. The interaction includes amino acid residues participating in the binding of RBD to the ACE2 receptor (Fig. 1b) (SI Table 2), including Arg403, Tyr489, Phe456, Leu455, Gln493, Tyr505, Tyr449, Gln498, Arg439, and Asn487. Therefore, it is predicted that these ligands will interfere with RBD binding to the ACE2 receptor.

**Table 1:** The binding of ligand (viii) is not affected by structural changes of RBD induced by the protein–binding partner.

Ligand	WT-RBD with ACE2 ΔG (Kd) PDB code 7c8d			WT-RBD with nanobody ΔG (Kd) PDB code 7kgj			WT-RBD with antibody ΔG (Kd) PDB code 7f63		
	R1	R1-2	R2	R1	R1-2	R2	R1	R1-2	R2
*i*	–	–	− 8.2 (1.6)	–	–	− 7.1 (9.5)	–	–	− 8.3 (1.3)
*ii*	–	− 7.9 (2.6)	–	–	− 7.2 (8.0)	–	–	− 7.2 (8.0)	–
*iii*	–	− 7.8 (3.0)	–	–	− 7 (11.2)	–	–	− 7.2 (8.0)	–
*iv*	–	− 8.1 (1.6)	–	–	− 7.7 (3.6)	–	–	− 7.6 (4.2)	–
*v*	–	− 7.3 (6.8)	–	− 7.4 (5.8)	–	–	–	− 7.5 (4.9)	–
*vi*	− 7.1 (9.5)	–	–	− 7.4 (5.8)	–	–	− 6.9 (13.1)	–	–
*vii*	–	–	− 7.3 (6.8)	− 7.3 (6.8)	–	–	− 6.8 (15.4)	–	–
*viii*	− 8 (2.2)	–	–	− 8 (2.2)	–	–	− 8.6 (0.8)	–	–

ΔG values are in kCal/mol and K_d_ values are in µM. (i) (1R,5R,6R,13R,21R)-16-[(1R,5R,6R,7R,13S,21R)-5,13-bis(3,4-dihydroxyphenyl)-6,9,17,19,21-pentahydroxy-4,12,14-trioxapentacyclo[11.7.1.02,11.03,8.015,20]henicosa-2(11),3(8),9,15,17,19-hexaen-7-yl]-5,13-bis(3,4-dihydroxyphenyl)-4,12,14-trioxapentacyclo[11.7.1.02,11.03,8.015,20]henicosa-2(11),3(8),9,15,17,19-hexaene-6,9,17,19,21-pentol; (ii) CinnamtanninB2; (iii) Escin Ia; (iv) Escin IIa; (v) Escin IIb; (vi) α-amyrin; (vii) β-amyrin; (viii) (1R)-1,6,7-trimethoxy-9-(4-methoxyphenyl)-8-[(4S)-1,4,9-trimethoxy-3-(4-methoxyphenyl)-5,6-dihydro-4H-phenalen-2-yl]-2,3-dihydro-1H-phenalene.

### MOD revealed new ligands of interest

**Figure 2:**
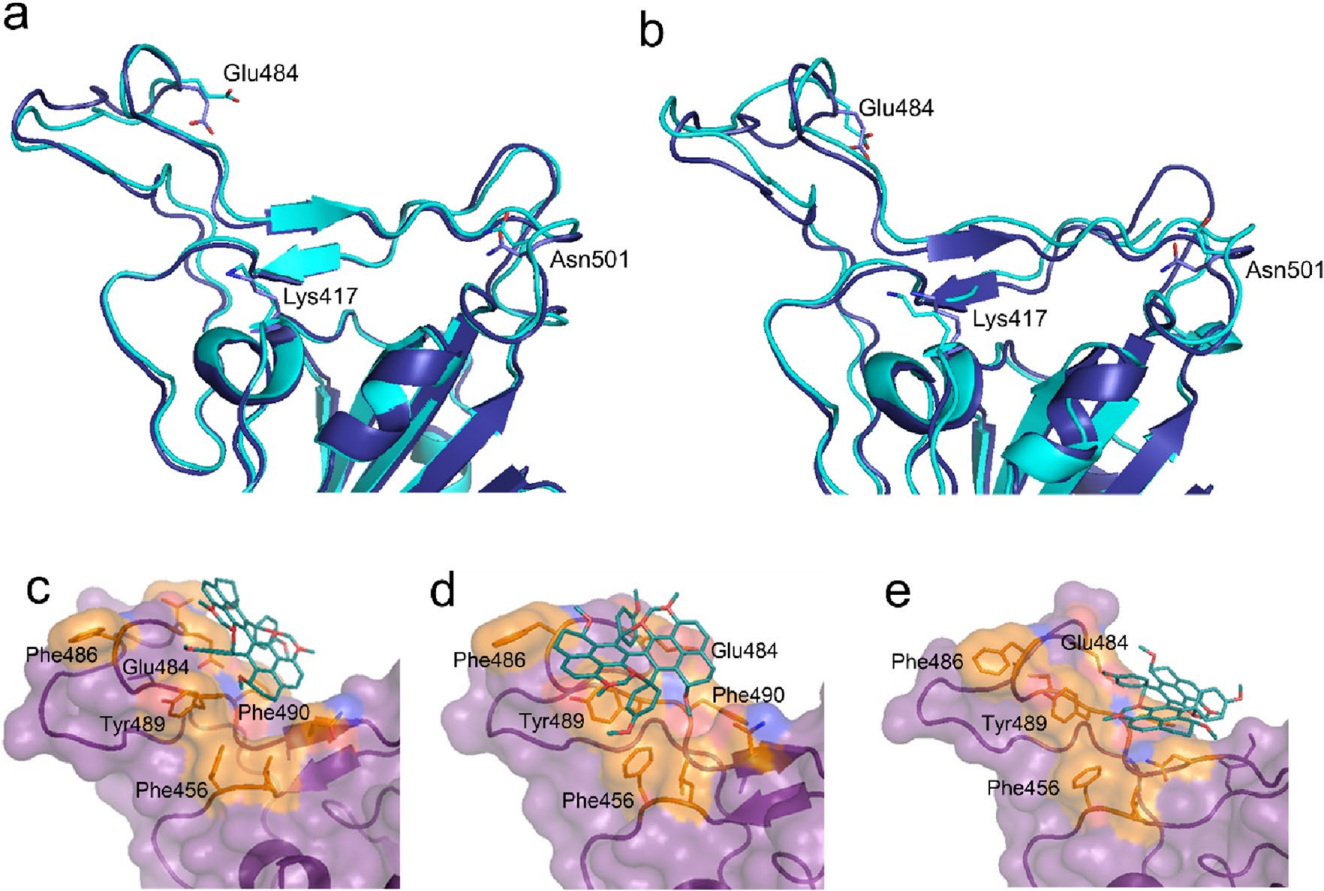
Predicting the effect of structural changes of RBD induced by protein partner reveals a ligand of interest. (**a**) Pairwise alignment of the structure of RBD solved in the presence of ACE2 (dark blue) (PDB Code: 7C8D) with that of RBD solved in the presence of nanobody (PDB Code: 7KGJ) (cyan) and (**b**) antibody (cyan) (PDB Code: 7F63). (**c**) The predicted binding pocket of ligand 8 using the RBD structure solved in the presence of ACE2, (**d**) nanobody, or (**e**) antibody.

The RBD structure is widely used for molecular docking studies and discovering ligands interfering with the RBD-binding to the ACE2 receptor^12,14–22^. The structures determined both using X-ray diffraction and Electron microscopy have been used. The structure of RBD by itself has never been solved due to its instability and the structure is always solved in the presence of one of its binding-protein partners namely ACE2 receptor, antibody, or nanobody. The binding partner is removed to prepare the structure for in silico studies. However, it has never been tested if solving the structure in the presence of different protein partners will affect the results of in silico screening. We first compared the structure of WT-RBD solved in the presence of ACE2, antibody, or nanobody (Fig. 2a, b). The results showed conformational changes and differences between these structures, specifically in the docking surface used for in silico screenings. Although the backbone structure is practically conserved, the orientation of side chains at the RBD surface are different. We hypothesized that these conformational changes could affect the outcome of in silico screening and that if the binding of a ligand was not affected by the conformational changes, that ligand would be ideal as a therapeutic lead molecule. Therefore, we performed molecular docking studies using all the 70 NPs, and the structure of WT-RBD solved in the presence of ACE2, antibody or nanobody (Methods) (SI Table 1). As an example, the results for the top eight ligands (smallest K_d_) are summarised in Table 1. For most of the ligands including those tested previously such as β-amyrin^40^, either the binding pocket shifted between R1 and R2 or the dissociation constant increased (binding affinity reduced). This observation is likely due to the conformational changes induced by ACE2, antibody, or nanobody binding to the RBD (Fig. 2a, b). Surprisingly, the binding energy and the binding pocket of two ligands (*ligands vi* and *viii*) were not affected by the conformational changes of RBD (Table 1). *Ligand viii* has the smallest dissociation constant and, thus, the highest binding affinity (Table 1). In all the structures, *ligand viii* continues to interact with amino acid residues in the R1 pocket of RBD (Fig. 2c–e). The amino acid residues of this pocket (SI Table 2) are among the key residues interacting with the ACE2 receptor (Fig. 1b). These results strongly suggest that *ligand viii* will interfere with the RBD-binding to the ACE2 and is a lead molecule for designing and synthesizing new inhibitors of viral entry to host cells.

### Screening using multiple RBD variants revealed its vulnerable pocket

**Figure 3:**
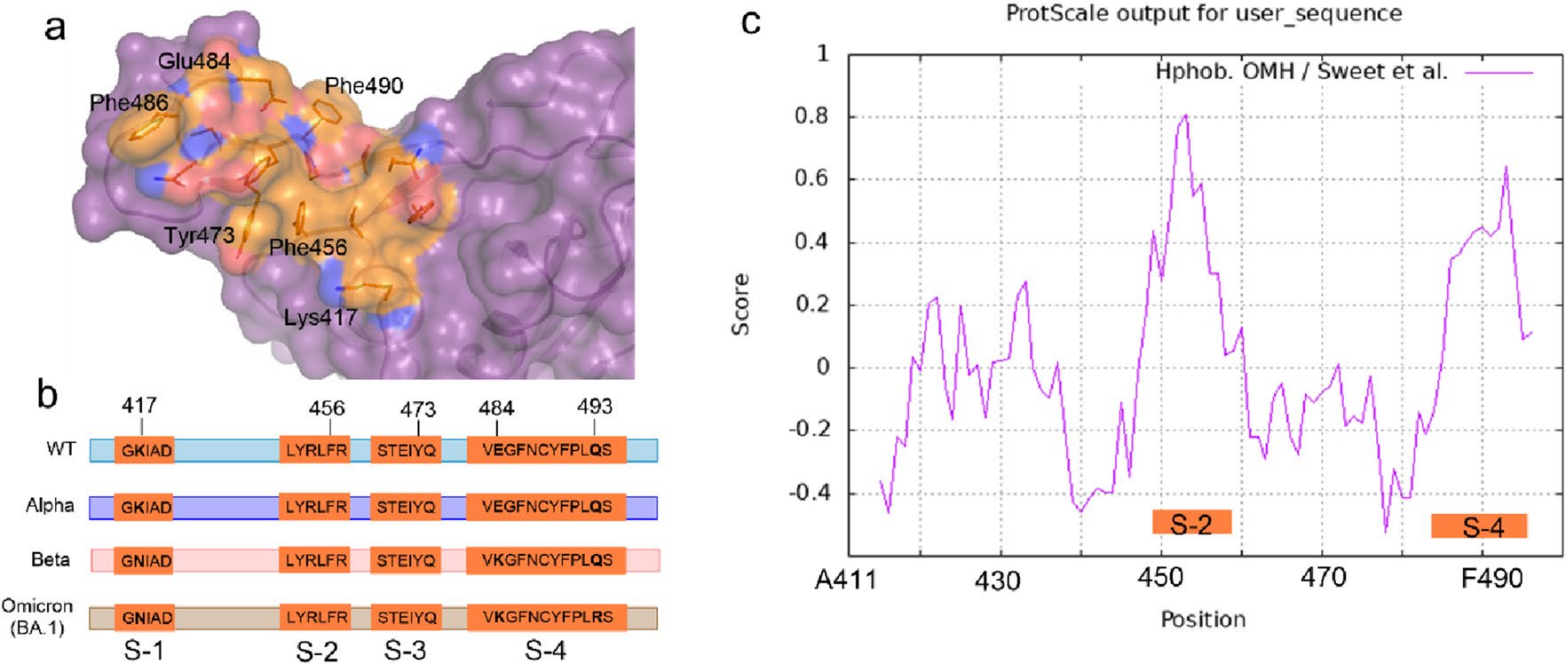
The vulnerable site of RBD has two conserved hydrophobic segments. (**a**) The structure of the vulnerable R1 region of RBD. (**b**) The vulnerable region consists of four short peptides (S-1 to S-4). These peptides are conserved. (**c**) Analysis of the peptides shows that peptide S-2 and S-4 have highly conserved hydrophobic regions.

Some of the emerging variants of SARS-CoV-2 can escape antibodies generated by the immune response. This escape phenomenon is confirmed by several previous studies^44–47^. We showed that mutations like N501Y on RBD abolish the binding of NPs of commensal microbiota like bile acids and minimize the interference of these NPs with the RBD binding to the ACE2 receptor^20^. We proposed a new mechanism of selection and evolution of the virus^20^. These observations highlight the growing need for discovering broad-spectrum small molecule inhibitors of RBD-binding to the ACE2 receptor. To predict which of the eight NPs of interest can bind to the R1 or R2 pocket of all major variants (alpha, beta, and omicron) and inhibit RBD-binding to ACE2, we used molecular docking studies taking advantage of RBD structures from all major variants. The ΔG values and the binding pocket of each ligand obtained from PyRx are given in Table 2. Mutations of RBD significantly increased the dissociation constant (reduced binding affinity) and shifted the binding pocket of all ligands except that of *ligand viii*. This ligand continued to interact with amino acid residues of R1 pocket with high affinity (Fig. S3). Therefore, R1 pocket is potentially an Achilles heel of RBD and ideal for structural-guided design of small molecule inhibitors of RBD binding to the ACE2 receptor and viral entry. This predicted pocket is different that the previously reported vulnerable epitopes of RBD targeted by various neutralizing antibodies^27–31^. This pocket consists of four short peptides (Fig. 3a, b). We used ProtScale server and Hphob.OHH scale^48^ and analyzed the hydrophobicity of R1 pocket encompassing these peptides (Fig. 3c). The results revealed that S-2 and S-4 peptides are in two hydrophobic regions, and there is no or little variability among the major variants in the amino acid sequences of these hydrophobic peptides.

**Table 2:** The ligand (viii) binding pocket and dissociation constant are not affected by mutations of RBD.

Ligand	WT ΔG (K_d_)			Alpha ΔG (K_d_)			Beta ΔG (K_d_)			Omicron ΔG (K_d_)		
	R1	R1-2	R-2	R1	R1-2	R-2	R1	R1-2	R-2	R1	R1-2	R-2
*i*	–	–	− 8.2 (1.6)	–	–	− 7.2 (8.0)	–	− 7.1 (9.5)	–	–	− 6.8 (15.4)	–
*ii*	–	− 7.9 (2.6)	–	–	− 6.7 (18.2)	–	–	− 7.1 (9.5)	–	–	− 7.2 (8.1)	–
*iii*	–	− 7.8 (3.0)	–	–	–	− 7.1 (9.5)	–	− 7 (11.2)	–	–	–	− 7.1 (9.5)
*iv*	–	− 8.1 (1.6)	–	–	–	− 7.4 (5.8)	–	− 7.2 (8.1)	–	–	–	− 7.4 (5.8)
*v*	–	− 7.3 (6.8)	–	–	–	− 7.4 (5.8)	–	− 6.9 (13.1)	–	–	–	− 7.3 (6.8)
*vi*	− 7.1 (9.5)	–	–	–	–	− 6.8 (15.4)	− 7.7 (3.6)	–	–	–	− 6.9 (13.1)	–
*vii*	–	–	− 7.3 (6.8)	− 7 (11.2)	–	–	–	–	− 7.9 (2.3)	–	− 7.1 (9.5)	–
*viii*	-8 (2.2)	–	–	-8.5 (0.97)	–	–	− 8.9 (0.5)	–	–	− 8 (2.2)	–	–

ΔG values are in kCal/mol and K_d_ values are in µM. PDB codes for WT, Alpha, Beta and Omicron variants are 7C8D, 7NEG, 7PS0, and 7WBP, respectively.

### Predicting the molecular structure of ligands binding to the vulnerable region of RBD

**Figure 4:**
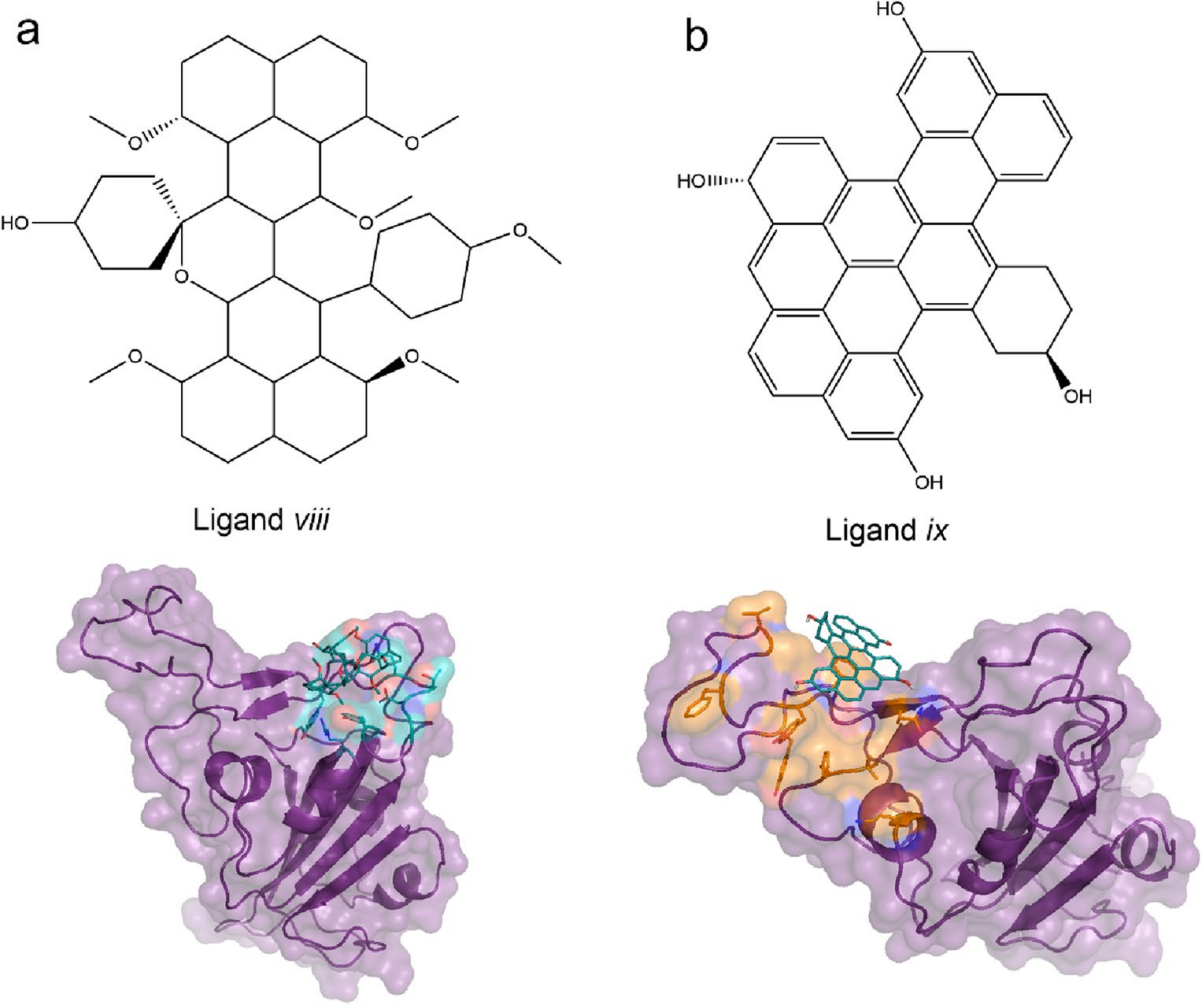
Structural requirement for designing ligands targeting vulnerable site of RBD. (**a**) The 2D chemical structure of ligand (viii) (top panel). After optimization the ligand folds and binds to R2 region with increased dissociation constant. (**b**) The chemical structure of the designed ligand (ix) (top panel). The optimized ligand has a flat surface, and it binds to the vulnerable R1 region of RBD. The docking results are from WT-RBD (PDB code 7C8D).

Based on in silico screening, we found that the presence of aromatic/aromatic hydroxyl groups is essential for strong binding to the RBD. By comparing the chemical structure of *ligand viii* with other ligands we hypothesized that the flat surface of the *ligand viii* is vital for its binding to the vulnerable R1 site. To test this prediction, we used density functional theory to optimize the structure of *ligand viii * (Fig. S3). The energy minimized structure of ligand *viii* bends around a central C7-C9 bond (Fig. S3). Next, we performed molecular docking using the optimized structure of *ligand viii*. The results show that after optimization the dissociation constant of the optimized *ligand viii* increased between 6- and 85-fold (Table 3). Additionally, the binding pocket shifted towards the R2 pocket on the RBD surface (Fig. 4a). To confirm that the significant increase in the dissociation constant and the shift in the binding pocket result from the bent conformation of the ligand, we virtually designed and optimized a new ligand (*ligand ix*) (Figs. 4b and S3) with a flat surface as in the original structure of *ligand viii*. The molecular structure of *ligand ix* is very similar to that of the hypericin^49^, a NP with antiviral activity against SARS-CoV-2. We then performed molecular docking using *ligand ix*. The results show that our designed ligand *ix* binds to the predicted vulnerable site of RBD (Fig. 4b) in all the structures with a dissociation constant at least ten-fold (Table 3) less than those obtained for the ligand *viii* in its original conformation (Table 2). In all the structures tested, we found that *ligand ix* binds to the R1 and slides over this pocket (Fig. S4). *Ligand ix* has a flat surface and multiple benzene and phenol rings. Similarly, in silico and biochemical screening have shown that several drugs with multiple benzene and phenol rings, like Hydroxycamptothecine^50^, KT203^51^, BMS195614^51^, and rilapladib^52^, interact with RBD and interfere with its binding to the ACE2 receptor. The binding energies of *ligand ix *(Table 3) are significantly smaller than those of drugs, such as KT203 (− 8.73 kCal/mol) BMS195614 (− 8.25 kCal/mol), obtained using PyRx^51^.

**Table 3:** A designed ligand with tight binding to the vulnerable site of RBD.

Ligand	WT (ACE2) ΔG (K_d_)			WT (Nanobody) ΔG (K_d_)			WT (antibody) ΔG (K_d_)			Alpha ΔG (K_d_)			Beta ΔG (K_d_)			Omicron ΔG (K_d_)		
	R1	R1-2	R-2	R1	R1-2	R-2	R1	R1-2	R-2	R1	R1-2	R-2	R1	R1-2	R-2	R1	R1-2	R-2
Optimized *viii*	–	–	− 6.9 (13.1)	–	− 5.4 (151)	–	–	− 5.3 (178)	–	–	− 5.6 (109)	–	–	− 6.1 (48)	–	–	–	− 5.6 (109)
Optimized *ix*	− 10.5 (0.04)	–		− 9.8 (0.12)	–	–	− 9.2 (0.31)	–	–	− 9.3 (0.26)	–	–	− 9.3 (0.26)	–	–	− 9.6 (0.16)	–	–

ΔG values are in kCal/mol and K_d_ values are in µM.

### Molecular dynamics revealed conformational stability of ligands

**Figure 5:**
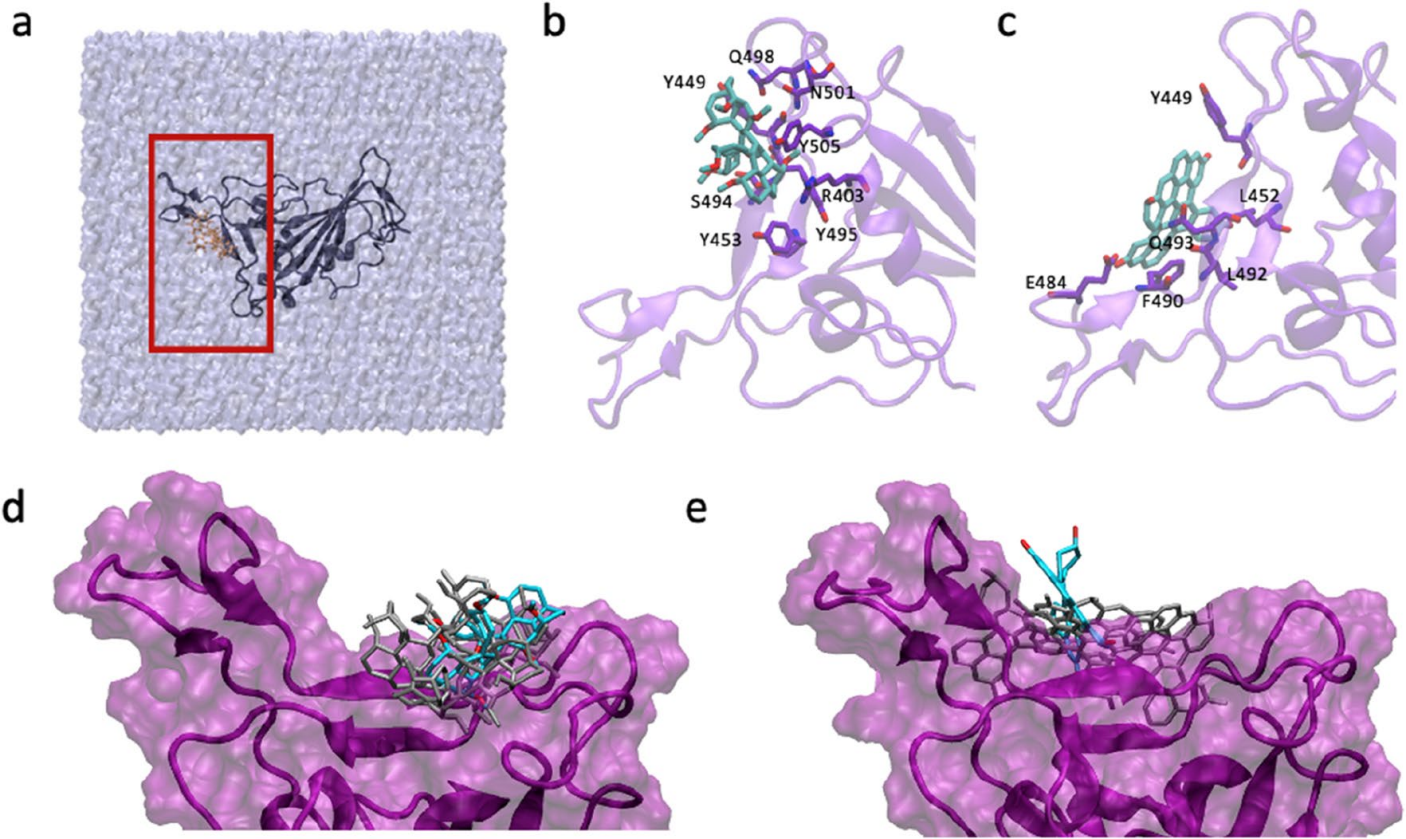
Results from classical molecular dynamics simulations for WT-RBD model using 7c8d.pdb structure. (**a**) Simulation box consisting of RBD protein, ligand (ix depicted in orange) and 78,310 water molecules (ice-cube representation), red box indicate the RBM of the RBD of the SARS-CoV-2 Spike protein. (**b**) Close up into the binding of ligand *viii* to R2 site of WT-RBD in conformation with strongest interaction (snapshot after 99.0 ns, − 33.9 kcal/mol), (**c**) binding of ligand *ix* to R1 site of RBD in conformation with strongest interaction (snapshot after 93.3 ns, − 30.9 kcal/mol). Ligands are shown as cyan sticks while relevant RBD residues interacting with the ligands are highlighted as purple sticks. Conformational space of ligand *viii* (**d**) and ligand *ix* (**e**) sampled over the MD simulation. Only representative ligand conformations characterized by highest interaction energies with WT-RBD are depicted as grey stick.

To obtain a molecular understanding of the ligand docking site and its dynamic properties, we carried out a series of classical nanosecond-scale molecular dynamics (MD) simulations. Specifically, we investigated the conformational stability and flexibility of the two docked ligands (*ligands viii* and *ix*) in the R1 and R2 binding pockets of WT RBDs and the variants, as well as the nature and strength of the intermolecular interactions. In total, six models were considered for these computations (see “Methods”). First, the RBD binding pocket accommodates both *ligands viii* and *ix*, irrespective of the initial starting structure used for the molecular simulations and the mutations of the RBD in the SARS-CoV-2 variants. The binding of these ligands is not restricted to the specific sites in R2 and R1 (Fig. 4), respectively, but to various regions of R1 and R2 (Figs. 5 and S6). While *ligand viii* binds to both R2 and R1, *ligand ix* slides over R1 pocket (Fig. S6). This observation is consistent with the results of rigid docking showing that *ligand viii* binding pocket changes while *ligand ix* binds to different sites of pocket R1 in different structures (Fig. S6). The amino acid residues of RDB interacting with *ligand viii* and *ix* are summarized in supplementary table 6. Among these residues, Arg403, Tyr449, Gln498, Asn501, Glu484, Phe490, Gln493, Tyr453 directly interact with ACE2 receptor. Therefore, consistent with the MOD studies *ligands viii* and *ix* are predicted to interfere with RBD binding to the ACE2.

The extent of conformational changes which undergo the structural models upon thermal relaxation can be estimated by monitoring the evolution of root-mean-square deviations of atomic positions (RMSD) experienced by all heavy atoms with respect to the initial reference structure during the simulation. In our analysis, the initial reference structure is represented by the corresponding crystal structure with the DFT geometry optimized docked ligand in the pose as predicted by the in silico docking experiments (vide supra). Another measure for protein compactness and stability is the radius of gyration (RG). The flexibility of the ligands and their binding environment can be accessed by evaluating the root-mean-square fluctuations (RMSF) of each heavy atom around their average position during the MD trajectory. Furthermore, the solvent-accessible protein surface (SASA) is used to determine whether the ligand is retained inside the binding pocket, or it expels out from the binding cavity. These quantities were computed over the last 10 ns of MD simulations (considering all three repetitions), when all systems reached a thermal equilibrium (Fig. S5). The average RMSD and RMSF values are listed in Table 4.

**Table 4:** Structural properties of the proteins, the ligands viii and ix, and the protein–ligand complexes: average root mean square deviations (RMSD) of heavy atoms of ligands *viii* and *ix* and their corresponding docking sites with respect to the initial structural models of WT-RBD and the variant, average root mean square fluctuations (RMSF) of heavy atoms of the ligands and their docking sites, radius of gyration (RG) of the proteins, solvent accessible surface area (SASA) of the ligands and the protein–ligand complex and average number of contacts between them.

Ligand *viii*						
	WT-RBD with ACE2 PDB code 7c8d	WT-RBD with nanobody PDB code 7kgj	WT-RBD with antibody PDB code 7f63	RBD Alpha variant PDB code 7neg	RBD Beta variant PDB code 7ps0	RBD Omicron variant PDB code 7wbp
Protein						
RMSD	1.08 $\pm$ 0.06	0.94 $\pm$ 0.11	1.61 $\pm$ 0.12	1.13 $\pm$ 0.08	1.21 $\pm$ 0.06	1.01 $\pm$ 0.07
RMSF	0.47 $\pm$ 0.28	0.48 $\pm$ 0.34	0.60 $\pm$ 0.39	0.48 $\pm$ 0.32	0.46 $\pm$ 0.31	0.47 $\pm$ 0.33
RG	18.2 $\pm$ 0.02	18.2 $\pm$ 0.02	19.2 $\pm$ 0.02	17.6 $\pm$ 0.02	18.2 $\pm$ 0.02	18.2 $\pm$ 0.02
Ligand						
RMSD	0.86 $\pm$ 0.08	1.85 $\pm$ 0.06	2.09 $\pm$ 0.23	1.88 $\pm$ 0.06	2.53 $\pm$ 0.07	1.86 $\pm$ 0.06
RMSF	0.28 $\pm$ 0.20	0.27 $\pm$ 0.18	0.27 $\pm$ 0.17	0.27 $\pm$ 0.18	0.29 $\pm$ 0.18	0.27 $\pm$ 0.17
SASA	870.5 $\pm$ 12.2	879.5 $\pm$ 13.9	892.5 $\pm$ 13.0	887.0 $\pm$ 12.0	892.6 $\pm$ 11.0	884.7 $\pm$ 12.6
Protein–Ligand						
Number of contacts	28.2 $\pm$ 5.5	15.7 $\pm$ 5.3	10.8 $\pm$ 3.1	15.1 $\pm$ 5.2	17.5 $\pm$ 5.4	17.1 $\pm$ 4.6
SASA	11,024.8 $\pm$ 66.1	10,978.9 $\pm$ 90.1	12,623.8 $\pm$ 98.4	10,733.0 $\pm$ 96.9	11,433.1 $\pm$ 92.2	11,367.1 $\pm$ 83.3

Ligand* ix*						
	WT-RBD with ACE2 PDB code 7c8d	WT-RBD with nanobody PDB code 7kgj	WT-RBD with antibody PDB code 7f63	RBD Alpha variant PDB code 7neg	RBD Beta variant PDB code 7ps0	RBD Omicron variant PDB code 7wbp
Protein						
RMSD	1.33 $\pm$ 0.11	1.02 $\pm$ 0.11	1.16 $\pm$ 0.10	1.05 $\pm$ 0.08	0.81 $\pm$ 0.07	0.95 $\pm$ 0.12
RMSF	0.48 $\pm$ 0.33	0.47 $\pm$ 0.33	0.51 $\pm$ 0.34	0.45 $\pm$ 0.28	0.44 $\pm$ 0.27	0.46 $\pm$ 0.32
RG	18.1 $\pm$ 0.02	18.2 $\pm$ 0.02	19.2 $\pm$ 0.02	17.6 $\pm$ 0.02	18.2 $\pm$ 0.02	18.2 $\pm$ 0.02
Ligand						
RMSD	0.29 $\pm$ 0.06	0.28 $\pm$ 0.05	0.25 $\pm$ 0.06	0.27 $\pm$ 0.07	0.31 $\pm$ 0.06	0.26 $\pm$ 0.06
RMSF	0.17 $\pm$ 0.08	0.17 $\pm$ 0.08	0.17 $\pm$ 0.08	0.18 $\pm$ 0.09	0.17 $\pm$ 0.08	0.18 $\pm$ 0.09
SASA	682.5 $\pm$ 10.6	704.6 $\pm$ 7.9	722.5 $\pm$ 7.9	718.4 $\pm$ 10.8	724.3 $\pm$ 8.4	715.5 $\pm$ 9.9
Protein–Ligand						
Number of contacts	15.1 $\pm$ 3.8	13.7 $\pm$ 3.6	19.8 $\pm$ 4.0	13.5 $\pm$ 3.6	10.0 $\pm$ 3.6	12.5 $\pm$ 3.9
SASA	10,992.1 $\pm$ 72.9	10,680.5 $\pm$ 63.3	12,125.6 $\pm$ 85.1	10,467.5 $\pm$ 72.0	11,258.3 $\pm$ 73.4	11,304.9 $\pm$ 73.6

All these quantities were evaluated over the last 10 ns of the corresponding MD trajectories (including repetitions). The number of contacts was computed using Numpy 1.20.3, and MDAnalysis 2.0.0^13^ with Python 3.8 compatible packages. A contact is defined as an event where the distance between ligand and protein is below 3 Å. SASA and RG were computed using VMD^57^ tools. The units for RMSD, RMSF and RG values is Å, while SASA is given in Å^2^.

The relatively low (around 1.0 Å) RMSD and RG values predicted for the six protein–ligand structural models suggest minor conformational changes of the RBD upon ligand binding, regardless of the nature of the ligand. Interestingly a slightly higher RMSD value of 1.61 Å is predicted when *ligand viii* binds the crystal structure of WT RBD complexed to the antibody. This may be a consequence of the initial arrangement of the side chain atoms in 7f63.pdb structure, in particular the Gln493 side chain, which points away from Ser494. In addition, the RMSF values of the RBD residues at the ligand docking site is relatively low (c.a 0.5 Å) for all models suggesting that the RBD mutations in Alpha-, Beta- and Omicron- SARS-CoV-2 variants practically do not affect the conformational mobility at the receptor binding site. The most flexible residues at the RBD were identified as Gln498 and Phe497. These residues flip their side chains towards the ligands upon docking. Other flexible side chains interacting with the ligands were Arg403, TYyr505 and Gln493 (Fig. 5).

The dynamic properties of the two ligands, however, are significantly different. While *ligand viii* shows high flexibility as reflected by RMSD values up to 2.5 Å, and RMSF values below 0.3 Å, *ligand ix* is very rigid (RMSD < 0.3 Å, RMSF < 0.2 Å) (Fig. S6). In particular, the methoxycyclohexane-moiety in *ligand viii* shows the highest mobility as reflected by the different conformations it adopts when docking onto the protein surface (Table S4) and by the fluctuations of the dihedral angle τ_C2-C10-C22-C31_ (Table S4 and Fig. S3) of more than 5° predicted during the MD simulations. The opposite is observed for *ligand ix,* which practically slides over the R1 surface as a rigid body without changing its conformation (Fig. S6). Thus, *ligand viii* is more prone to adapt its form to the RBD topology compared to *ligand ix*. Indeed, analysis of atomic contact for neighbouring atoms of binding pocket supports such a statement showing that *ligand viii* remains in very close contact with the S-protein during the course of all MD simulations while *ligand ix* interacts with the protein less than 95% of the simulation time. Moreover, the number of contacts established between ligand and RBD are correlated with ligand flexibility: the flexible *ligand viii* forms in average 18 contacts with side chains of RBD whereas the rigid *ligand ix* only 14 contacts with it. Specifically, *ligand viii* presents short-range contacts of hydrophobic and H-bond nature with Arg403, Tyr495, Gln498, Asn501, Tyr505, Tyr453, Ser494 and Tyr449. Ligand *ix* mainly interacts with Tyr449, Leu452, Leu492, Phe490, Gln493 and Glu484 (Fig. 5).

The strength of the interactions between *ligands viii* and *ix* with RBD and their nature has been quantified by computing the Van der Waals and electrostatic contributions of the average total interaction energy (Table 5). Among the two ligands, *ligand ix* shows the strongest interaction with R1 site of wild-type RBD as well as with alpha, beta and omicron variants of the SARS-CoV-2 as reflected by the predicted low average interaction energies of − 26.5 kcal/mol, − 19.9 kcal/mol, − 25.5 kcal/mol and − 25.9 kcal/mol, respectively. These values are followed by ligand *viii*, which also shows moderate interaction energy with the R2 site. Furthermore, the computations clearly indicate that Van der Waals forces are the primary contributor to the interaction between the ligands and the RBD. Electrostatics contribute to less than 1% of *ligand viii* binding to the RBD and only 15% to the binding of *ligand ix* to the RBD. Glu484 is responsible for the attractive electrostatic interactions in R1.

**Table 5:** Average total interaction energy (kcal/mol) between optimized ligands *viii* and *ix* with binding sites of wild type RBD and the variant of SARS-CoV-2 virus.

Ligand	WT (ACE2)			WT (Nanobody)			WT (antibody)			Alpha			Beta			Omicron		
	MD1	MD2	MD3	MD1	MD2	MD3	MD1	MD2	MD3	MD1	MD2	MD3	MD1	MD2	MD3	MD1	MD2	MD3
Optimized *viii*	− 30.0 $\pm 1.9$	− 29.6 $\pm 2.0$	− 29.8 $\pm 2.3$	− 17.1 $\pm 4.6$	− 16.1 $\pm 2.5$	− 12.1 $\pm 4.1$	− 12.7 $\pm 3.6$	− 17.7 $\pm 2.3$	− 15.8 $\pm \mathrm{3.6}$	− 12.9 $\pm 4.2$	− 17.3 $\pm 3.8$	− 17.2 $\pm 3.3$	− 22.2 $\pm 2.0$	− 22.1 $\pm 2.1$	− 20.7 $\pm 3.4$	− 19.7 $\pm 1.7$	− 14.2 $\pm 3.2$	− 13.5 $\pm 2.8$
Optimized *ix*	− 24.5 $\pm 3.1$	− 21.3 $\pm 2.6$	− 25.8 $\pm 3.1$	− 23.8 $\pm 2.3$	− 23.7 $\pm 2.0$	− 25.0 $\pm 3.7$	− 32.7 $\pm 2.5$	− 32.0 $\pm 2.3$	− 30.0 $\pm 2.3$	− 22.2 $\pm 2.2$	− 14.9 $\pm 2.4$	− 22.7 $\pm 3.9$	− 24.7 $\pm 2.7$	− 26.6 $\pm 2.5$	− 25.1 $\pm 2.8$	− 26.3 $\pm 2.8$	− 24.0 $\pm 2.3$	− 27.3 $\pm 2.2$

All energy values are evaluated over the last 10 ns of the MD trajectories (3 repetitions).

From the standpoint of SASA, *ligand ix* shows a SASA value of about 700 Å^2^, which is almost 200 Å^2^ lower than the value predicted for *ligand viii,* indicating that *ligand ix* is less exposed to water and a larger part of its surface is protected by the receptor protein, compared to *ligand viii.* Thus, the highly mobile *ligand viii* is stabilized via short, weak interactions with several side chains in the entire RBD. On the other hand, the rigid *ligand ix* establishes strong hydrophobic interactions with specific residues of the vulnerable R1 pocket. Furthermore, the sequence mutations detected in alpha, beta and omicron variants of the SARS-CoV-2 practically do not affect the binding properties of *ligands viii* and *ix* to RBD. These results support the predictions from the in silico rigid docking experiments described above.

## Conclusions

In silico screening of small molecule ligands is a powerful tool to identify the suitable lead molecules helping the process of drug discovery^10,11,53^. These studies take advantage of the structure(s) of a protein. In the case of SARS-CoV-2 RBD, the structure is always solved in the presence of a binding partner like ACE2, antibody, or nanobody. Thus, the binding partner is removed from the structure before in silico screening. However, how the interaction of the binding partner with RBD affects the outcome of molecular docking has not been investigated^12,14–22^. To address this question, we used the structure of WT-RBD solved in the presence of ACE2, antibody, or nanobody (Table 1). We used MOD and screened the binding of more than 70 NPs. Our findings show that the structural changes of WT-RBD induced by the binding of ACE2, antibody, or nanobody significantly affect the binding of most ligands. These studies enabled us to identify a vulnerable binding pocket (R1) of RBD, where binding of a ligand was not affected by the structural changes of RBD and mutations observed in the emerging variants. A combination of MOD and MD simulations allowed us to define the structural requirement of ligands targeting the R1 pocket. We found that hydroxyl groups, the flat surface and the rigid structure of the ligand are essential for binding to R1 pocket. To confirm this, we designed *ligand ix*. Molecular docking and dynamics studies confirmed that *ligand ix* binds to the R1 pocket irrespective of the structural changes induced by ACE2, antibody, or nanobody and the mutations present in alpha, beta, and omicron variants. The *ligand ix* structure is very similar to the antiviral and anticancer NP hypericin. Recent biochemical studies revealed that hypericin reduces SARS-CoV-2 infectivity via different mechanisms^54^ and inhibits the S-glycoprotein binding to the ACE2 receptor^26^.

In silico studies have limitations. Virtual screening methods can generate false positives/negatives. These false outcomes can arise from inaccuracies of the structural models used, inclusion/exclusion of water molecules in the models, and approximation of the binding sites^55^. Our data suggest that MOD reduces the generation of false positive/negatives outcomes. Although we used the structure of multiple variants of SARS-CoV-2, new variants of the virus continue to emerge. Given the adaptability of the virus and its continuous evolution and possible mutational landscapes, it is not possible to screen the binding of ligands against all possible variants. Therefore, further experimental studies and screening using multiple variants of the virus are required to corroborate the findings of our in silico screening. A limitation of our study is the sample size of the structures used. We selected one structure either determined using X-ray diffraction or electron microscopy (SI Methods). Screening using more structures, at least 3 structures solved using X-ray and 3 structure solved using electron microscopy for each RBD will extend the confidence of MOD studies and potentially identifying broad-spectrum lead molecules for drug discovery and development. Finally, in silico studies cannot predict off-target and toxicity of newly identified compounds. It is possible that like hypericin, which has some cytotoxicity and can inhibit protein kinase C (PKC)^56^, our predicted *ligand ix* will have some cellular toxicity and off-target activity. These activities and IC50 value of the ligand must be determined experimentally to validate its potential for further drug development.

In summary, we demonstrate that the application of MOD improves identification of ligands binding to various RBD variants and structures irrespective of mutations in the RBD and structural variants. The MOD findings combined with MD calculations reveal new insight into the structural requirement of designing SARS-CoV-2 viral entry inhibitors. Further experiments using a larger number of structures of RBD should be conducted to corroborate our findings. We suggest that MOD should be used to help structural-guided design and synthesis of new lead molecules for drug discovery and development.

### Supplementary Information

Supplementary Information.

## Data Availability

All data generated or analysed during this study are included in this published article and its supplementary information files.

## Abbreviations

ACE2: Angiotensin-converting enzyme 2
MD: Molecular dynamics
MOD: Multi-structural molecular docking
NP: Natural products
PKC: Protein kinase C
RBD: Receptor-binding domain
TMPRSS2: Transmembrane serine protease 2
WT: Wild-type

## References

[1] Ciotti M (2020). The COVID-19 pandemic. Crit. Rev. Clin. Lab. Sci..

[2] Hoffman M (2020). SARS-CoV-2 cell entry depends on ACE2 and TMPRSS2 and is blocked by a clinically proven protease inhibitor. Cell.

[3] Yan R (2020). Structural basis for the recognition of SARS-CoV-2 by full-length human ACE2. Science.

[4] Jackson CB, Farzan M, Chen B, Choe H (2022). Mechanisms of SARS-CoV-2 entry into cells. Nat. Rev. Mol. cell Biol..

[5] Li W (2003). Angiotensin-converting enzyme 2 is a functional receptor for the SARS coronavirus. Nature.

[6] Ferraz, M., Moreira, E., Coêlho, D. F., Wallau, G. & Lins, R. Immune evasion of SARS-CoV-2 variants of concern is driven by low affinity to neutralizing antibodies. *Chem. Comm.* **57**, 6094–6097 (2021). 10.1039/d1cc01747k 34037640

[7] Gupta RK (2021). Will SARS-CoV-2 variants of concern affect the promise of vaccines?. Nat. Rev. Immunol..

[8] Hu J (2022). Increased immune escape of the new SARS-CoV-2 variant of concern Omicron. Cell. Mol. Immunol..

[9] Maher MC (2022). Predicting the mutational drivers of future SARS-CoV-2 variants of concern. Sci. Transl. Med..

[10] Terstappen GC, Reggiani A (2001). In silico research in drug discovery. Trends Pharmacol. Sci..

[11] Seifert MHJ, Wolf K, Vitt D (2003). Virtual high-throughput in silico screening. Biosilico.

[12] Nag A, Paul S, Banerjee R, Kundu R (2021). In silico study of some selective phytochemicals against a hypothetical SARS-CoV-2 spike RBD using molecular docking tools. Comput. Biol. Med..

[13] Muratov EN (2021). A critical overview of computational approaches employed for COVID-19 drug discovery. Chem. Soc. Rev..

[14] Singh R, Bhardwaj VK, Sharma J, Kumar D, Purohit R (2021). Identification of potential plant bioactive as SARS-CoV-2 Spike protein and human ACE2 fusion inhibitors. Comput. Biol. Med..

[15] Rout J, Swain BC, Tripathy U (2022). In silico investigation of spice molecules as potent inhibitor of SARS-CoV-2. J. Biomol. Struct. Dyn..

[16] Behloul N (2021). In silico identification of strong binders of the SARS-CoV-2 receptor-binding domain. Eur. J. Pharmacol..

[17] Muhseen ZT, Hameed AR, Al-Hasani HMH, Tahir-ul-Qamar M, Li G (2020). Promising terpenes as SARS-CoV-2 spike receptor-binding domain (RBD) attachment inhibitors to the human ACE2 receptor: Integrated computational approach. J. Mol. Liq..

[18] Deganutti G, Prischi F, Reynolds CA (2021). Supervised molecular dynamics for exploring the druggability of the SARS-CoV-2 spike protein. J. Comput. Aided. Mol. Des..

[19] Gangadevi S (2021). Kobophenol A inhibits binding of host ACE2 receptor with spike RBD domain of SARS-CoV-2, a lead compound for blocking COVID-19. J. Phys. Chem. Lett..

[20] Drageli J, Mroginski MA, Ebrahimi KH (2021). Hidden in plain sight: Natural products of commensal microbiota as an environmental selection pressure for the rise of new variants of SARS-CoV-2. ChemBioChem.

[21] Honarmand Ebrahimi K (2020). SARS-CoV-2 spike glycoprotein-binding proteins expressed by upper respiratory tract bacteria may prevent severe viral infection. FEBS Lett..

[22] Yepes-Pérez AF, Herrera-Calderon O, Quintero-Saumeth J (2022). Uncaria tomentosa (cat’s claw): A promising herbal medicine against SARS-CoV-2/ACE-2 junction and SARS-CoV-2 spike protein based on molecular modeling. J. Biomol. Struct. Dyn..

[23] Yang J (2021). Computational design and modeling of nanobodies toward SARS-CoV-2 receptor binding domain. Chem. Biol. Drug Des..

[24] Yang J (2021). Structure-based discovery of novel nonpeptide inhibitors targeting SARS-CoV-2 Mpro. J. Chem. Inf. Model..

[25] Carino A (2020). Hijacking SARS-CoV-2/ACE2 receptor interaction by natural and semi-synthetic steroidal agents acting on functional pockets on the receptor binding domain. Front. Chem..

[26] Mohamed FF (2022). Hypericum perforatum and its ingredients hypericin and pseudohypericin demonstrate an antiviral activity against SARS-CoV-2. Pharmaceuticals.

[27] Li T (2021). Uncovering a conserved vulnerability site in SARS-CoV-2 by a human antibody. EMBO Mol. Med..

[28] McCallum M (2021). N-terminal domain antigenic mapping reveals a site of vulnerability for SARS-CoV-2. Cell.

[29] Joyce MG (2020). A cryptic site of vulnerability on the receptor binding domain of the SARS-CoV-2 spike glycoprotein. BioRxiv.

[30] Makdasi E (2021). Neutralizing monoclonal anti-SARS-CoV-2 antibodies isolated from immunized rabbits define novel vulnerable spike-protein epitope. Viruses.

[31] Burnett DL (2021). Immunizations with diverse sarbecovirus receptor-binding domains elicit SARS-CoV-2 neutralizing antibodies against a conserved site of vulnerability. Immunity.

[32] Trott O, Olson AJ (2023). AutoDock Vina: improving the speed and accuracy of docking with a new scoring function, efficient optimization, and multithreading. J. Comput. Chem..

[33] Dallakyan, S. & Olson, A. J. Small-molecule library screening by docking with PyRx. In *Chemical Biology* 243–250 (Springer, 2015). 10.1007/978-1-4939-2269-7_19 25618350

[34] Dragelj J, Mroginski MA, Ebrahimi KH (2021). Hidden in plain sight: Natural products of commensal microbiota as an environmental selection pressure for the rise of new variants of SARS-CoV-2. ChemBioChem.

[35] Kieseritzky G, Knapp E-W (2008). Optimizing pKA computation in proteins with pH adapted conformations. Proteins Struct. Funct. Bioinforma..

[36] Wolf A (2020). The redox-coupled proton-channel opening in cytochrome c oxidase. Chem. Sci..

[37] Vanommeslaeghe K (2010). CHARMM general force field: A force field for drug-like molecules compatible with the CHARMM all-atom additive biological force fields. J. Comput. Chem..

[38] Jo S, Kim T, Iyer VG, Im W (2008). CHARMM-GUI: A web-based graphical user interface for CHARMM. J. Comput. Chem..

[39] Dragelj J, Mroginski MA, Ebrahimi KH (2021). Hidden in plain sight: Natural products of commensal microbiota as an environmental selection pressure for the rise of new variants of SARS-CoV-2. ChemBioChem.

[40] Wahyuni DK (2022). Molecular simulation of compounds from n-hexane fraction of *Sonchus arvensis* L. leaves as SARS-CoV-2 antiviral through inhibitor activity targeting strategic viral protein. J. Pharm. Pharmacogn. Res..

[41] Eskandari V (2022). Repurposing the natural compounds as potential therapeutic agents for COVID-19 based on the molecular docking study of the main protease and the receptor-binding domain of spike protein. J. Mol. Model..

[42] Pandya M (2022). Unravelling Vitamin B12 as a potential inhibitor against SARS-CoV-2: A computational approach. Inform. Med. Unlocked.

[43] Dinata R (2023). Repurposing immune boosting and anti-viral efficacy of Parkia bioactive entities as multi-target directed therapeutic approach for SARS-CoV-2: exploration of lead drugs by drug likeness, molecular docking and molecular dynamics simulation methods. J. Biomol. Struct. Dyn..

[44] Harvey WT (2021). SARS-CoV-2 variants, spike mutations and immune escape. Nat. Rev. Microbiol..

[45] Cao Y (2022). Omicron escapes the majority of existing SARS-CoV-2 neutralizing antibodies. Nature.

[46] Hoffmann M (2021). SARS-CoV-2 variants B.1.351 and P.1 escape from neutralizing antibodies. Cell.

[47] Zhou D (2021). Evidence of escape of SARS-CoV-2 variant B.1.351 from natural and vaccine induced sera. Cell.

[48] Sweet RM, Eisenberg D (1983). Correlation of sequence hydrophobicities measures similarity in three-dimensional protein structure. J. Mol. Biol..

[49] Liu Q (2020). Hypericin: Single molecule spectroscopy of an active natural drug. J. Phys. Chem. A.

[50] Tsegay KB (2021). A repurposed drug screen identifies compounds that inhibit the binding of the COVID-19 spike protein to ACE2. Front. Pharmacol..

[51] Choudhary S, Malik YS, Tomar S (2020). Identification of SARS-CoV-2 cell entry inhibitors by drug repurposing using in silico structure-based virtual screening approach. Front. Immunol..

[52] Alexpandi R, De Mesquita JF, Pandian SK, Ravi AV (2020). Quinolines-based SARS-CoV-2 3CLpro and RdRp inhibitors and Spike-RBD-ACE2 inhibitor for drug-repurposing against COVID-19: an in silico analysis. Front. Microbiol..

[53] Davies JW, Glick M, Jenkins JL (2006). Streamlining lead discovery by aligning in silico and high-throughput screening. Curr. Opin. Chem. Biol..

[54] Delcanale P (2022). The interaction of hypericin with SARS-CoV-2 reveals a multimodal antiviral activity. ACS Appl. Mater. Interfaces.

[55] Phatak SS, Stephan CC, Cavasotto CN (2009). High-throughput and in silico screenings in drug discovery. Expert Opin. Drug Discov..

[56] Kocanova S (2006). Characterization of the interaction of hypericin with protein kinase C in U-87 MG human glioma cells. Photochem. Photobiol..

[57] Humphrey W, Dalke A, Schulten KVMD (1996). Visual molecular dynamics. J. Mol. Graph..

