# Supplementary material for: Multi-structural molecular docking (MOD) combined with molecular dynamics reveal the structural requirements of designing broad-spectrum inhibitors of SARS-CoV-2 entry to host cells
Source: Sci Rep. 2023 Sep 29;13:16387. doi: 10.1038/s41598-023-42015-2 (PMC10541870; doi:10.1038/s41598-023-42015-2)
Supplement: Supplementary file 1 — Supplementary Information. [file 41598_2023_42015_MOESM1_ESM.pdf]

Supplementary data

Multi-structural molecular docking (MOD) combined with molecular dynamics calculates reveal the structural requirements of designing broad-spectrum inhibitors of SARS-CoV-2 entry to host cells

Anqi Da[1], Meritxell Wu-Lu[2], Jovan Dragelj[2], Maria Andrea Mroginski[2], Kourosh H. Ebrahimi[1]

[1] Institute of Pharmaceutical Science, King's College London, London, UK

[2] Institute of Chemistry, Technische Universität Berlin, Berlin, Germany

Correspondance:

Kourosh.ebrahimi@kcl.ac.uk

andrea.mroginski@tu-berlin.de

SI Methods.

Selection of Natural Products (NPs) (Ligands). In our previous study, a limited number of metabolites and NPs produced by commensal microbiota were tested[1]. Of these ligands some bile acids showed reasonable binding affinities to the RBD. A common similarity among bile acids is that they all comprise aromatic and aromatic hydroxyl groups[1]. Hence, we continued the previous study to test more metabolites and NPs from different sources searching for NPs with multiple aromatic rings. Because research suggests that honey[2,3], dragon fruit[4] and extract of Aesculus hippocastanum[5] may help treat Covid-19, we tested some of their known NPs. In addition to these known sources, we introduced one new plant into our study, *Pontederia crassipes*, which had never been studies in treatments of Covid-19 before. *Pontederia crassipes*, also known as water hyacinth, is one of the most invasive weed species in the world. The rapid reproductive capacity enables it to put stress on the local ecosystem and causes significant adverse economic and ecological impacts [31]. Despite its side effects, we unexpectedly found that many of its natural products comprise multiple aromatic and aromatic hydroxyl groups, which might be inhibitors for SARS-CoV-2 RBD binding to the ACE2 receptor. Hence, we selected several NPs of *Pontederia crassipes* to perform molecular docking. We used PubChem website (PubChem (nih.gov)) to search and select NPs.

Selection of RBDs (Macromolecules). The original SARS-CoV-2 RBD from Wuhan, also defined as wide type (WT) RBD was selected as the macromolecule for molecular docking test. However, the RBD by itself is not stable. Hence, the structure of RBD is always solved in the presence of its binding partners like ACE2 receptor, antibody, or nanobody. In addition, mutations on the RBD structure may affect the NPs binding. Hence, to study the effects of conformational changes and mutations on ligand binding, we selected the structure of three WT-RBD solved in the presence of ACE2, antibody or nanobody. In addition, three RBD from the main variants, namely Alpha, Beta and Omicron variant RBD were chosen. The structure of RBDs used in our research can be found in Protein Data Bank (PDB) by codes 7C8D, 7KGJ, 7F63 (structures of WT-RBD in complex with ACE2, a synthetic nanobody Sb45 and a neutralizing antibody chAb-45, respectively), and 7NEG, 7PS0, and 7WBP (Alpha variant in complex with COVOX-296 Fab, Beta variant in complex with beta-24 Fabs and Omicron variant in complex with ACE2, respectively). Numerous structures have been determined using X-ray diffraction and electron microscopy with various resolutions (4-2 Å). To have a representation of all these various structures in our screening, we selected 3 structures determined using X-ray diffraction and 3 structures determined using electron microscopy with a resolution ranging from 3.9-2.19 Å.

Structure	Method	Resolution (Å)
7c8d	Electron microscopy	3
7f63	Electron Microscopy	3.9
7kgj	X-ray Diffraction	2.3
7neg	X-ray Diffraction	2.19
7ps0	X-ray Diffraction	2.92
7wbp	electron microscopy	2.79

Visualization and analysis of molecular dockings data. Modes of ligands obtained from docking studies using Pyrx were later added to the RBD structure using PyMol software. The interaction between RBD and ligands were analysed and highlighted by showing the amino acids associated and surface changes of RBD taking advantages of PyMol.

Supplementary Table 1. Predicted ΔG values of the ligands tested in this study. ΔG values are in kCal/mol.

Group	Lignad	WT-RBD with ACE2 ΔG PDB code 7c8d			WT-RBD with Nanobody ΔG PDB code 7kgj			WT-RBD with Antibody ΔG PDB code 7f63		
		R1	R1-2	R2	R1	R1-2	R2	R1	R1-2	R2
Aflatoxin	aflatoxinB1	---	---	-6.8	-6.8	---	---	-6	---	---
	aflatoxinB2	---	---	-6.9	---	-6.1	---	-5.9	---	---
	aflatoxinG1	---	-6.8	---	-7.7	---	---	-5.9	---	---
	aflatoxinM1	---	---	-6.8	-6.7	---	---	-6.2	---	---
	aflatoxinG2-13C17	---	-6.7	---	-5.7	---	---	-6.7	---	---
	AmorphinFlavonoid	---	-7.5	---	-6.5	---	---	---	-6.5	---
	Alkaloid ND-305B	---	---	-7	-7.3	---	---	---	-6.4	---
Aesculus hippocastanum	Isoescin v	---	-7.3	---	-6.1	---	---	---	-6.8	---
	Procyanidin C1	---	-8	---	-6.8	---	---	---	-7.2	---
	(1R,5R,6R,13R,21R)-16-[(1R,5R,6R,7R,13S,21R)-5,13-bis	---	-8.2	---	---	-7.1	---	---	-8.3	---
	3,3',3'',4,4',4''-Hexahydro-2alpha,2'alpha,2''alpha-tris(3	---	---	-7.4	-6.8	---	---	-6.8	---	---
	cinnamtanninA2	---	---	-7.3	---	-6.2	---	---	-6.6	---
	cinnamtanninB2	---	-7.9	---	---	-7.2	---	-7.2	---	---
	escinIb	---	-7.2	---	---	-6.9	---	---	-7	---
	escinIa	---	-7.8	---	---	-7	---	---	-7.2	---
	escinIIa	---	-8.1	---	---	-7.7	---	---	-7.6	---
	escinIIb	---	-7.3	---	---	-7.4	---	---	-7.5	---
	escinIIIb	---	-7.1	---	---	-6.9	---	---	-7	---
	escinIV	---	-7.2	---	---	-6.8	---	---	-7.2	---
	escinV	---	-7.3	---	---	-6.7	---	---	-7	---
choline	CDP-choline	---	-6.5	---	---	-5.4	---	---	-5.5	---
	Cholie-alforscerate	---	-4.4	---	-5.1	---	---	-4.7	---	---
	acetylcholine	---	-3.6	---	-4.2	---	---	-3.6	---	---
	betaine	---	-3.2	---	-3.7	---	---	-3.3	---	---
	lysophosphatidylcholine	---	-4.9	---	-4.3	---	---	---	-4.3	---
	sphingomyelin	---	-4.5	---	-4.5	---	---	-4.2	---	---
honey compounds	alpha_maltulose	---	---	-5.7	-4.7	---	---	-5.7	---	---
	D-fructose	---	-4.7	---	-5.3	---	---	-5.2	---	---
	D-glucose	---	-5	---	---	-4.5	---	-5.2	---	---
	erlose	---	-5.8	---	---	-5.5	---	-5.6	---	---
	isomaltose	---	-5.7	---	-6.4	---	---	-6.1	---	---
	kojibiose	---	---	-5.4	-5.8	---	---	-5.3	---	---
	turanose	---	-5.4	---	-6.3	---	---	-5.3	---	---
	proline	---	---	-4	-4.9	---	---	-4.2	---	---
	gluconicacid	---	-4.5	---	-5.2	---	---	-5	---	---
	gluconolactone	---	---	-4.7	-5.2	---	---	-5.1	---	---
	Riboflavin	---	---	-6.5	-6	---	---	-6.1	---	---
	Niacin	---	-4.8	---	-3.8	---	---	-3.9	---	---
	Folic acid	---	-6.7	---	---	-6.4	---	---	---	-7
	Pantothenic acid	---	---	-4.9	-4.7	---	---	---	---	-5
	Vitamin B6	---	-4.4	---	-4.3	---	---	-4.2	---	---
	Ascorbic acid	---	-5	---	---	---	-5.4	---	---	-5.1
	Pinocembrin	---	---	-6.3	-6	---	---	-5.8	---	---
	Citric acid	---	-4.8	---	---	---	-5.5	---	-4.3	---
	Succinic acid	---	-4.3	---	---	---	-4.9	---	-3.5	---
	Pyroglutamic acid	---	---	-4.3	---	---	-5.3	---	---	-4.6
dragon fruit	copperlinoleate	---	---	-4.9	-4.9	---	---	-3.7	---	---
	Oleic acid	---	---	-4.3	-3.5	---	---	-3.9	---	---
	palmiticacid	---	-4.3	---	-4.8	---	---	-3.6	---	---
	1-Hexadecyne	---	-4.2	---	-3.6	---	---	-3.1	---	---
	2-Chloroethyl linoleate	-4.5	---	---	---	-4	---	---	-4	---
	1-nonadecene	-4.2	---	---	-4.1	---	---	-3.4	---	---
	6-tetradecanesulfonicacid	---	-4.6	---	---	-3.7	---	-4.3	---	---
	octacosane	-4.2	---	---	-4.2	---	---	-3.2	---	---
	Hexadecyltrichloroacetate	---	-4.2	---	-4.7	---	---	-3.7	---	---
	2-(((2-Ethylhexyl)oxy)carbonyl)benzoicacid	---	-5.2	---	-4.1	---	---	-4.8	---	---
	Campesterol	-6.2	---	---	-6	---	---	-5.7	---	---
	squalene	---	-5.4	---	-5.1	---	---	-4.6	---	---
	Stigmasterol	-6.8	---	---	-6.6	---	---	-6.4	---	---
	Phthalicacid6-ethyloct-3-yl2-ethylhexylester	---	-5.2	---	---	-4.5	---	-4.5	---	---
	1，2-Benzenedicarboxylicacidmono(2-ethylhexyl)este	---	-5.5	---	---	-4.6	---	-4.8	---	---
	alpha-Amyrin	-7.1	---	---	-7.4	---	---	-6.9	---	---
	Ergosta-468(14)22-tetraen-3-one	-7.1	---	---	---	-7	---	-6.9	---	---
	Stigmast-4-en-3-one	-6.1	---	---	-5.7	---	---	-6	---	---
	beta-amyrin	---	---	-7.2	-7.3	---	---	-6.8	---	---
	gamma-sitosterol	-6.5	---	---	-6.2	---	---	-5.9	---	---
	beta-sitosterol	-6.4	---	---	-6.2	---	---	-5.7	---	---
Pontederia crassipes	(1R)-1,6,7-trimethoxy-9-(4-methoxyphenyl)-8-[(4S)-1,4	-8	---	---	-8	---	---	-8.6	---	---
	(1R)-1,6,7-trimethoxy-9-(4-methoxyphenyl)-8-[(4S)-2,4	---	-7.7	---	-6.5	---	---	-6.9	---	---
	CID_163195957	---	---	-5.9	---	-8.9	---	-7.5	---	---
	Apigenin7-(6-malonylglucoside)	---	---	-7.1	---	-7.8	---	---	-6.3	---
	Delphinidin3-glucosylglucoside	---	---	-6.4	---	-5.9	---	---	-6	---

The molecule highlighted in orange is ligand *viii*. Some of the ligands are predicted to bind to the RBD and shown to have antiviral activity against SARS-CoV-2. These ligands are: β-amyrin[15], folic acid[16], vitamin B6[17], steroid such as campesterol and stigmasterol[18].

Supplementary Table 2. The amino acid residues forming the binding pocket of the eight selected ligands.

Ligand	Structure	Residues
i	7c8d	ASN501, THR500, CLN498, TYR449, SER494, GLN493, TYR453, ARG403
	7f63	CYS488, SER494, GLU484, TYR449, GLN493, PHE490, LEU492, TYR489
	7kgj	CYS488, SER494, GLU484, TYR449,GLN493, PHE490
	7neg	GLN493, LYS417, ARG403, TYR505,TYR501,TYR495
	7ps0	TYR449, PHE490, ARG403, GLN493, TYR501
	7wbp	HIS505, ARG403, TYR501, ARG498, SER496, TYR495, TYR449, ARG493, SER494
ii	7c8d	GLY416, GLN409, SER494,GLN493, TYR453, GLN498, TYR449, ARG403, TYR505, ASN501, GLU406, LYS417
	7f63	SER494, LEU492, PHE490, GLU484, TYR449, TYR489
	7kgj	TYR449, ASN450, GLN493, LEU492, GLU484, CYS488, PHE486, SER494, LEU452
	7neg	TYR489, GLN493, SER494, TYR501, GLN498, TYR449, TYR495, GLN498, PHE456
	7ps0	GLN493, LEU492, PHE490, TYR489, CYS488, LYS484, GLY485, PHE486
	7wbp	ARG493, TYR453, SER494, ARG403, HIS505, SER496, TYR501, ARG498, PHE490, TYR449
iii	7c8d	ARG403, TYR505, ASN501, GLN498, GLY496, GLN493, TYR453, GLU406, LYS417
	7f63	ARG408, GLU406, ASP405, TYR505, GLN409, TYR453, ARG403, THR415, LYS417
	7kgj	GLU484, GLN493, SER494, PHE490, TYR489
	7neg	TYR505, TYR501, ARG403, TYR453, GLN493, SER494, TYR449, GLN498
	7ps0	ARG408, GLN409, ASN417, TYR421, ASP405, ARG403, TYR505, TYR453
	7wbp	HIS505, TYR501, ARG403, TYR453, SER496, TYR449, ARG498, ARG403
iv	7c8d	LYS419, TYR453, ARG403,TYR505, GLY496,LEU455, GLN493, GLN498, ASN501
	7f63	ARG403, TYR495, SER494, GLN493, LEU492, PHE490, GLU484 TYR489(phobic),CYS488,GLN498,TYR505
	7kgj	GLU484, GLN493, SER494, PHE490, PHE456, TYR489, LEU492, TYR449
	7neg	TYR501, TYR449, GLN498, TYR495, TYR453, GLN493, TYR505, SER494, GLY496, SER494
	7ps0	TYR489, TYR449, PHE490, LYS484, PHE456, LEU455, GLN493, SER494, LEU492, PRO491
	7wbp	GLY502, HIS505, ARG403, TYR495, SER494, TYR453, SER496, TYR449, TYR501,THR500,ARG493,ARG498
v	7c8d	TYR453, GLN493, SER494, ARG403, TYR505, ASN501, GLY496, TYR495
	7f63	GLU484, PHE490, GLN493, LEU492, SER494, ARG403, GLY496, TYR489
	7kgj	GLU484, PHE490, GLN493, SER494, CYS488, PHE456, TYR489, LEU452, TYR449
	7neg	GLN493, TYR453, SER494, TYR495, TYR501, GLN498, TYR505, GLY496
	7ps0	LYS484, PHE490, GLN493, LEU492, GLN493, PHE490, SER494, TYR449
	7wbp	TYR453, ARG403, HIS505, GLY502, TYR449, SER494, TYR495, SER496, ARG498, TYR501
vi	7c8d	TYR489, GLU484, PHE490, PHE456, LEU455, GLN493
	7f63	TYR489, PHE490,SER494,LEU492, LEU452, GLN493, PHE456,
	7kgj	SER494, TYR489,PHE490, PHE456, LEU455, GLU484, GLN493, LEU492
	7neg	TYR505, TYR501, TYR495, GLN498, TYR449, GLU493, SER494
	7ps0	PHE490, TYR489, PHE456, LEU455, GLN493, LEU492, LYS484
	7wbp	ARG493, SER496, TYR449, TYR495, HIS505, ARG403
vii	7c8d	TYR505, THR500, GLN498, TYR495, TYR453, ARG403
	7f63	PHE456, GLN493, TYR489, PHE490, GLU484, PHE456, LEU492
	7kgj	SER494, PHE490, TYR489, PHE456, GLN493, LEU452, LEU455, TYR489, GLU484
	7neg	GLU484, PHE486, TYR489, PHE456, LEU455, GLN493, GLU484
	7ps0	SER494, GLN493, TYR453, TYR501, TYR505, ARG403, TYR495, SER494
	7wbp	HIS505, ARG403, TYR453, ARG493, TYR449, SER494, 496, TYR501, TYR495
viii	7c8d	GLY485, CYS488, PHE490,GLU484, TYR489, GLN493
	7f63	PHE490, LEU492, SER494, GLU484, TYR489, GLU493, PHE456
	7kgj	GLY485, GLU484, PHE490,TYR489, PHE456, PHE486, LEU455, GLN493
	7neg	TYR473, ARG457, TYR421, PHE456, LYS417,LEU455, TYR489,TYR421,LYS458
	7ps0	GLY485, GLN493, PHE490,TYR489, LYS455, PHE456, TYR489, PHE490, LYS484, PHE486
	7wbp	PHE490, ARG493, TYR449, LEU452, SER494

Supplementary Table 3a. CHARMM compatible force field parameters for bonded interaction computed for ligand ***viii***. Atomic partial (RESP) charges are given together with the ligand structure in figure S1.

**BONDS**						
**CG301**	OG3C61	360.00	1.4	150		
**ANGLES**						
**CG311**	CG301	OG3C61	45.50	114.50		
**CG321**	CG301	OG3C61	45.50	114.50		
**CG311**	CG311	OG3C61	45.00	111.50		
**CG301**	OG3C61	CG311	95.00	109.70		
**DIHEDRALS**						
**OG3C61**	CG301	CG311	CG311	0.8000	3	0.00
**OG3C61**	CG301	CG311	HGA1	0.1600	3	0.00
**OG3C61**	CG301	CG321	CG321	0.8000	3	0.00
**OG3C61**	CG301	CG321	HGA2	0.1600	3	0.00
**CG311**	CG301	OG3C61	CG311	0.5300	1	180.00
**CG311**	CG301	OG3C61	CG311	0.6800	2	0.00
**CG311**	CG301	OG3C61	CG311	0.2100	3	180.00
**CG311**	CG301	OG3C61	CG311	0.1500	4	0.00
**CG321**	CG301	OG3C61	CG311	0.5300	1	180.00
**CG321**	CG301	OG3C61	CG311	0.6800	2	0.00
**CG321**	CG301	OG3C61	CG311	0.2100	3	180.00
**CG321**	CG301	OG3C61	CG311	0.1500	4	0.00
**CG311**	CG311	CG311	CG311	0.5000	4	180.00
**CG311**	CG311	CG311	OG301	0.1600	1	180.00
**CG311**	CG311	CG311	OG301	0.3900	2	0.00
**CG311**	CG311	CG311	OG3C61	0.1900	1	180.00
**CG311**	CG311	CG311	OG3C61	10.000	2	180.00
**CG311**	CG311	CG311	OG3C61	0.6000	3	0.00
**CG311**	CG311	CG311	OG3C61	0.0800	4	180.00
**OG3C61**	CG311	CG311	HGA1	0.1950	3	0.00
**CG321**	CG311	OG301	CG331	0.4000	1	0.00
**CG321**	CG311	OG301	CG331	0.4900	3	0.00
**CG311**	CG311	OG3C61	CG301	0.5300	1	180.00
**CG311**	CG311	OG3C61	CG301	0.6800	2	0.00
**CG311**	CG311	OG3C61	CG301	0.2100	3	180.00
**CG311**	CG311	OG3C61	CG301	0.1500	4	0.00
**HGA1**	CG311	OG3C61	CG301	0.1950	3	0.00

Supplementary Table 3b. CHARMM compatible force field parameters for bonded interaction computed for ligand *ix*. Atomic partial (RESP) charges are given together with the ligand structure in figure S1.

**BONDS**						
**CG2DC1**	CG311	365.00	1.50			
**ANGLES**						
**CG2DC1**	CG2DC1	CG311	48.00	123.50		
**CG311**	CG2DC1	HGA4	40.00	116.00		
**CG2DC1**	CG311	CG2R61	51.80	107.50		
**CG2DC1**	CG311	OG311	75.70	110.10		
**CG2DC1**	CG311	HGA1	45.00	111.50		
**DIHEDRALS**						
**CG2R61**	CG2DC1	CG2DC1	CG311	0.5605	1	180.00
**CG2R61**	CG2DC1	CG2DC1	CG311	69.692	2	180.00
**CG311**	CG2DC1	CG2DC1	HGA4	52.000	2	180.00
**CG2DC1**	CG2DC1	CG311	CG2R61	0.0000	3	180.00
**CG2DC1**	CG2DC1	CG311	OG311	19.000	1	180.00
**CG2DC1**	CG2DC1	CG311	OG311	0.4000	2	180.00
**CG2DC1**	CG2DC1	CG311	OG311	0.6000	3	180.00
**CG2DC1**	CG2DC1	CG311	HGA1	0.0300	3	0.00
**HGA4**	CG2DC1	CG311	CG2R61	0.2000	3	0.00
**HGA4**	CG2DC1	CG311	OG311	0.2000	3	0.00
**HGA4**	CG2DC1	CG311	HGA1	0.2000	3	0.00
**CG321**	CG2R61	CG2R61	CG321	24.000	2	180.00
**CG2R61**	CG2R61	CG311	CG2DC1	0.2300	2	180.00
**OG311**	CG311	CG321	CG2R61	18.000	1	180.00
**OG311**	CG311	CG321	CG2R61	0.3500	3	0.00
**CG2DC1**	CG311	OG311	HGP1	13.000	1	0.00
**CG2DC1**	CG311	OG311	HGP1	0.7000	2	0.00
**CG2DC1**	CG311	OG311	HGP1	0.1400	3	0.00
**CG2R61**	CG321	CG321	CG311	0.0400	3	0.00

Supplementary Table 4: Average dihedral angle $t_{C2\text{-}C10\text{-}C22\text{-}C31}$ (degree) of the cyclo-methoxy moiety of *ligand viii* docked to the 6 RBD models computed over the last 10 ns of the MD simulations (including repetitions).

DFT	7c8d	7kgj	7f63	7neg	7ps0	7wbp
**66.96**	74.61 ±6.55	47.92 ±6.54	49.81 ±7.01	48.53 ± 6.01	38.96 ±7.02	46.22 ±6.43

Supplementary Table 5. Cartesian coordinates (in Å) for the DFT optimized ligand structures.

M1	x	y	z		M2	x	y	z		M3	x	y	z
O	0.712	2.994	-2.380		O	0.567	3.112	-2.050		O	1.155	4.312	0.344
O	1.446	-2.935	-2.936		O	1.456	-2.770	-2.940		O	1.384	-3.692	-2.101
O	-2.764	-0.426	1.239		O	-2.963	-0.525	1.315		O	-2.165	-0.506	2.362
O	2.232	-0.403	0.309		O	2.145	-0.406	0.433		O	-4.307	0.790	3.013
O	-3.405	2.102	-0.898		O	-3.558	2.037	-0.710		O	2.792	-1.595	4.302
O	3.303	-1.643	3.122		O	3.086	-1.753	3.175		O	4.814	1.244	-2.509
O	5.353	0.462	-2.753		O	5.272	0.725	-2.524		O	-3.641	-0.769	-4.269
O	-4.854	-2.488	-4.120		O	-4.836	-2.423	-4.173		C	-0.795	2.919	0.382
C	-0.063	2.709	-0.094		C	-0.232	2.663	0.203		C	0.689	-2.931	0.059
C	0.693	-3.563	-0.717		C	0.699	-3.545	-0.771		C	-0.172	4.200	-0.177
C	-0.432	3.152	-1.530		C	-0.591	3.188	-1.208		C	0.486	-3.989	-1.011
C	0.891	-4.015	-2.182		C	0.920	-3.906	-2.257		C	-2.094	2.953	0.976
C	-1.173	3.066	0.965		C	-1.371	2.921	1.260		C	1.588	-3.192	1.139
C	2.069	-3.176	-0.126		C	2.061	-3.158	-0.146		C	-0.584	0.563	1.007
C	-0.675	0.167	0.176		C	-0.775	0.095	0.305		C	0.162	-0.740	1.020
C	-0.129	-1.266	0.558		C	-0.192	-1.342	0.619		C	-0.081	1.718	0.348
C	0.441	1.250	0.207		C	0.310	1.202	0.420		C	-0.018	-1.730	0.011
C	-0.496	-2.571	-0.313		C	-0.519	-2.609	-0.323		C	-0.959	5.463	0.197
C	-0.811	4.641	-1.529		C	-1.006	4.665	-1.117		C	0.747	-5.398	-0.466
C	1.813	-5.253	-2.195		C	1.870	-5.120	-2.333		C	-2.936	4.108	0.841
C	-1.826	4.480	0.790		C	-2.054	4.328	1.168		C	2.315	-4.425	1.222
C	3.071	-4.347	-0.159		C	3.085	-4.309	-0.231		C	-2.457	5.259	-0.007
C	-2.029	4.928	-0.667		C	-2.245	4.866	-0.260		C	2.152	-5.459	0.134
C	3.179	-4.942	-1.574		C	3.222	-4.818	-1.677		C	-2.592	1.805	1.684
C	-2.265	1.983	1.248		C	-2.441	1.799	1.444		C	1.747	-2.216	2.181
C	1.892	-2.576	1.285		C	1.861	-2.635	1.290		C	-1.784	0.627	1.708
C	-1.709	0.541	1.256		C	-1.852	0.379	1.377		C	1.205	1.608	-0.414
C	1.319	-1.160	1.096		C	1.251	-1.227	1.175		C	-0.990	-1.469	-1.100
C	1.755	0.744	-0.459		C	1.644	0.776	-0.262		C	-3.882	1.903	2.308
C	-1.686	-2.376	-1.303		C	-1.706	-2.390	-1.314		C	2.623	-2.524	3.265
C	-3.595	2.193	0.518		C	-3.766	2.025	0.709		C	-4.168	4.133	1.447
C	3.190	-2.671	2.140		C	3.146	-2.741	2.150		C	3.157	-4.656	2.289
C	-3.166	4.543	1.546		C	-3.407	4.309	1.903		C	-4.648	3.039	2.201
C	4.471	-3.900	0.357		C	4.469	-3.872	0.332		C	3.321	-3.706	3.316
C	-4.208	3.550	0.976		C	-4.416	3.335	1.247		C	2.442	1.443	0.234
C	4.436	-2.650	1.263		C	4.403	-2.657	1.282		C	1.200	1.649	-1.810
C	2.888	1.776	-0.332		C	2.748	1.827	-0.057		C	-2.369	-1.449	-0.858
C	1.623	0.267	-1.932		C	1.540	0.390	-1.763		C	-0.551	-1.249	-2.418
C	-2.515	-3.691	-1.436		C	-2.501	-3.715	-1.529		C	2.051	5.018	-0.490
C	-1.456	-1.773	-2.702		C	-1.478	-1.703	-2.675		C	1.018	-4.291	-3.330
C	0.420	3.015	-3.763		C	0.293	3.218	-3.432		H	-3.018	-0.329	2.801
C	1.530	-3.154	-4.329		C	1.560	-2.905	-4.343		C	3.621	1.328	-0.489
C	-3.375	-0.666	2.492		H	-2.633	-1.413	1.517		C	2.380	1.530	-2.556
H	-0.673	-1.496	1.478		H	-0.713	-1.639	1.539		C	-3.290	-1.223	-1.884
C	4.259	1.216	-0.767		C	4.138	1.331	-0.511		C	-1.456	-1.018	-3.448
C	2.888	0.532	-2.774		C	2.806	0.739	-2.572		C	3.599	1.369	-1.892
C	-3.399	-3.725	-2.701		C	-3.369	-3.699	-2.804		C	-2.834	-1.003	-3.188
C	-2.804	-1.343	-3.323		C	-2.831	-1.270	-3.281		C	-5.572	0.818	3.658
C	4.167	0.263	-1.974		C	4.083	0.452	-1.774		C	2.110	-1.939	5.518
C	-3.954	-2.340	-3.022		C	-3.958	-2.313	-3.054		C	4.845	1.248	-3.925
C	-4.595	1.835	-1.614		C	-4.732	1.780	-1.454		C	-5.041	-0.731	-4.059
C	2.549	-1.872	4.291		C	3.916	-2.007	4.287		H	-0.113	4.128	-1.276
C	5.673	-0.606	-3.614		C	5.625	-0.280	-3.447		H	-0.544	-3.924	-1.386
C	-5.658	-1.356	-4.364		C	-5.673	-1.304	-4.362		H	-0.755	5.680	1.252
H	-1.265	2.550	-1.921		H	-1.404	2.593	-1.648		H	-0.593	6.313	-0.390
H	-0.071	-4.296	-2.633		H	-0.031	-4.182	-2.734		H	0.632	-6.142	-1.263
H	0.063	5.204	-1.173		H	-0.151	5.224	-0.711		H	-0.007	-5.628	0.298
H	-1.001	4.974	-2.556		H	-1.188	5.059	-2.123		H	-3.015	6.170	0.241
H	1.936	-5.624	-3.220		H	2.014	-5.426	-3.376		H	-2.658	5.051	-1.070
H	1.311	-6.058	-1.642		H	1.380	-5.968	-1.835		H	2.875	-5.266	-0.670
H	-2.259	6.002	-0.674		H	-2.500	5.933	-0.201		H	2.372	-6.458	0.530
H	-2.889	4.416	-1.107		H	-3.084	4.364	-0.747		H	-4.799	5.013	1.342
H	3.697	-4.217	-2.215		H	3.732	-4.046	-2.267		H	3.706	-5.593	2.342
H	3.803	-5.846	-1.556		H	3.865	-5.708	-1.703		H	-5.621	3.107	2.672
H	-3.569	5.563	1.498		H	-3.832	5.321	1.919		H	3.992	-3.894	4.149
H	5.126	-3.695	-0.500		H	5.142	-3.629	-0.501		H	2.470	1.416	1.318
H	-4.972	3.359	1.740		H	-5.188	3.075	1.982		H	0.255	1.754	-2.337
H	5.326	-2.615	1.902		H	5.305	-2.630	1.906		H	-2.730	-1.611	0.153
H	2.631	2.636	-0.955		H	2.473	2.718	-0.627		H	0.512	-1.264	-2.628
H	0.775	0.747	-2.417		H	0.684	0.877	-2.228		H	1.764	6.072	-0.624
H	-1.861	-4.571	-1.441		H	-1.824	-4.576	-1.574		H	3.028	4.982	0.000
H	-0.779	-0.918	-2.664		H	-0.820	-0.837	-2.582		H	2.135	4.545	-1.480
H	0.047	3.991	-4.104		H	-0.429	2.454	-3.762		H	0.005	-3.990	-3.638
H	1.354	2.801	-4.288		H	-0.098	4.206	-3.714		H	1.735	-3.937	-4.077
H	-0.322	2.247	-4.032		H	1.238	3.059	-3.957		H	1.064	-5.390	-3.301
H	1.785	-2.194	-4.786		H	1.795	-1.913	-4.738		H	2.362	-1.161	6.242
H	2.306	-3.886	-4.595		H	2.357	-3.600	-4.642		H	1.025	-1.965	5.362

H	0.570	-3.499	-4.746	H	0.614	-3.249	-4.791	H	4.577	1.202	0.010
H	-2.635	-0.966	3.250	H	-1.242	0.136	-0.682	H	2.329	1.558	-3.638
H	-3.923	0.209	2.872	H	4.631	0.784	0.297	H	-4.350	-1.218	-1.654
H	-4.086	-1.485	2.348	H	2.811	1.802	-2.840	H	-1.118	-0.843	-4.465
H	-1.163	0.170	-0.801	H	-4.180	-4.432	-2.732	H	-5.683	-0.152	4.144
H	4.746	0.706	0.068	H	-3.126	-0.302	-2.858	H	-5.613	1.613	4.412
H	2.916	1.576	-3.109	H	-5.508	2.544	-1.292	H	-6.383	0.961	2.933
H	-4.228	-4.431	-2.579	H	-4.453	1.789	-2.511	H	5.893	1.125	-4.204
H	-3.070	-0.343	-2.959	H	-5.157	0.796	-1.202	H	4.259	0.419	-4.345
H	-5.047	0.883	-1.295	H	3.709	-1.225	5.023	H	4.470	2.195	-4.338
H	-5.345	2.632	-1.496	H	3.698	-2.989	4.740	H	-5.486	-0.527	-5.035
H	-4.326	1.766	-2.671	H	4.986	-1.975	4.036	H	-5.422	-1.690	-3.682
H	2.808	-2.837	4.759	H	4.887	-0.398	-4.256	H	-5.325	0.066	-3.359
H	2.790	-1.066	4.990	H	6.580	0.015	-3.892	H	2.449	-2.912	5.890
H	1.462	-1.862	4.109	H	5.750	-1.260	-2.955	C	1.029	-0.984	2.077
H	6.628	-0.361	-4.087	H	-6.278	-1.092	-3.464	O	1.181	0.004	3.000
H	5.785	-1.555	-3.062	H	-5.110	-0.393	-4.621	H	1.885	-0.285	3.619
H	4.922	-0.760	-4.405	H	-6.345	-1.543	-5.191				
H	-6.242	-1.073	-3.471	H	0.589	3.320	0.522				
H	-5.072	-0.480	-4.684	H	0.443	-4.519	-0.328				
H	-6.351	-1.622	-5.167	H	-0.829	2.922	2.216				
H	0.781	3.361	0.168	H	2.469	-2.326	-0.725				
H	0.417	-4.504	-0.218	H	0.589	1.230	1.486				
H	-0.611	3.119	1.908	H	-1.003	-3.266	0.410				
H	2.490	-2.383	-0.748	H	-1.388	5.035	1.684				
H	0.736	1.337	1.265	H	2.702	-5.140	0.385				
H	-0.993	-3.169	0.459	H	-2.763	1.887	2.491				
H	-1.136	5.204	1.247	H	1.100	-3.247	1.798				
H	2.686	-5.135	0.510	H	-1.337	0.230	2.343				
H	-2.557	2.163	2.293	H	1.168	-0.807	2.186				
H	1.115	-3.146	1.816	H	-2.389	-1.727	-0.768				
H	-1.171	0.432	2.215	H	-4.422	1.178	0.937				
H	1.255	-0.674	2.080	H	3.140	-3.733	2.637				
H	-2.344	-1.672	-0.780	H	-3.251	4.031	2.954				
H	-4.274	1.380	0.804	H	4.927	-4.723	0.852				
H	3.169	-3.641	2.668	H	-4.942	3.833	0.425				
H	-2.998	4.336	2.612	H	4.371	-1.718	0.721				
H	4.934	-4.736	0.898	H	2.788	2.112	1.002				
H	-4.739	4.002	0.131	H	1.373	-0.686	-1.825				
H	4.428	-1.733	0.668	H	-3.140	-3.884	-0.651				
H	2.948	2.126	0.706	H	-0.974	-2.383	-3.366				
H	1.428	-0.805	-1.925	H	4.769	2.196	-0.745				
H	-3.146	-3.793	-0.543	H	2.803	0.171	-3.510				
H	-0.974	-2.501	-3.358	H	-2.773	-3.981	-3.682				
H	4.910	2.048	-1.059	H	-2.720	-1.130	-4.364				
H	2.859	-0.093	-3.674	H	4.089	-0.611	-1.488				
H	-2.821	-4.071	-3.568	H	-4.556	-2.022	-2.173				
H	-2.701	-1.273	-4.414								
H	4.152	-0.780	-1.624								
H	-4.531	-1.983	-2.149								

M4	x	y	z	M7	x	y	z	M8	x	y	z
O	1.452	4.175	2.063	C	-4.476	-0.319	0.051	C	-4.479	2.063	1.859
O	1.796	-1.991	-2.100	C	2.727	0.635	-1.902	C	1.591	0.598	-2.243
O	-1.571	-1.139	-0.341	C	-3.845	-1.038	1.090	C	-3.610	1.295	2.821
O	3.081	-0.842	0.409	C	0.668	1.795	-2.709	C	-0.739	0.903	-3.048
O	-3.410	0.519	-1.568	C	0.129	2.909	-3.348	C	-1.665	1.181	-4.077
O	4.934	-2.498	2.455	C	-2.303	1.249	-3.202	C	-3.436	1.583	-2.325
O	7.091	1.427	-0.091	O	3.442	-5.454	0.296	O	4.582	-0.438	3.477
O	-5.697	-2.642	0.395	C	-3.757	0.276	-0.966	C	-4.100	1.877	0.416
C	0.705	2.315	0.762	C	-2.434	-1.177	1.068	C	-2.445	0.658	2.429
C	0.901	-3.615	-0.455	C	4.117	0.670	-1.777	C	2.994	1.043	-2.628
C	1.635	3.569	0.777	C	-1.765	-1.889	2.109	H	-4.460	3.135	2.115
C	1.041	-3.195	-1.935	C	2.095	1.635	-2.708	C	1.147	0.185	-0.986
C	-0.454	2.487	-0.274	C	-0.259	-0.734	-0.023	C	-1.690	-0.115	3.374
C	2.223	-4.101	0.249	C	0.501	-0.024	-0.994	C	0.653	0.797	-3.321
C	0.544	-0.233	0.286	C	-1.678	-0.553	0.035	C	-0.781	0.184	0.647
C	0.824	-1.510	1.102	C	1.894	-0.296	-1.126	C	-0.279	0.340	-0.695
C	1.392	0.893	0.905	C	-4.580	-1.609	2.178	C	-1.994	0.716	1.030
C	0.094	-2.770	0.574	C	4.905	1.563	-2.523	C	-1.198	1.286	-5.380
C	1.365	4.556	-0.377	C	-2.514	-2.377	3.224	C	-4.030	1.214	4.185
C	1.653	-4.375	-2.743	C	2.902	2.568	-3.440	C	3.456	0.329	-3.907
C	-0.050	2.921	-1.719	C	-3.943	-2.235	3.208	C	-2.179	-0.260	4.711
C	3.291	-4.638	-0.745	C	4.315	2.476	-3.366	C	1.098	0.919	-4.669
C	1.206	3.815	-1.713	C	-0.352	-2.076	2.062	C	-3.346	0.455	5.104
C	2.591	-5.303	-1.940	C	-0.164	0.876	-1.914	C	2.560	0.792	-5.053
C	-1.520	1.326	-0.252	C	0.378	-1.654	0.861	C	-0.522	-0.814	2.988
C	2.808	-3.125	1.335	C	0.925	3.788	-4.108	C	-1.174	0.783	-1.684
C	-0.967	-0.018	0.317	C	-2.333	0.179	-0.982	C	0.049	-0.654	1.589
C	2.358	-1.651	1.328	C	2.382	-1.488	-0.538	O	-2.122	1.523	-6.367
C	2.913	0.601	0.568	C	0.280	-2.596	3.200	C	-2.871	1.293	0.040
C	-1.420	-2.424	0.307	O	0.263	4.813	-4.726	C	2.050	-0.190	0.137
C	-2.232	1.318	-1.621	C	-1.846	-2.964	4.309	C	0.056	-1.723	3.869
C	4.336	-3.339	1.465	C	2.289	3.610	-4.179	C	-3.135	1.335	-3.784
C	0.043	1.735	-2.690	C	-0.459	-3.033	4.309	C	-1.519	-1.126	5.606
C	4.333	-3.565	-1.096	C	2.243	-3.439	0.984	C	0.168	1.140	-5.671
C	-1.296	0.953	-2.783	C	-1.559	0.812	-2.021	C	-0.435	-1.873	5.183
C	5.124	-3.138	0.161	C	1.646	-2.142	0.471	H	-1.659	1.609	-7.214
C	3.824	1.106	1.737	C	3.631	-2.214	-1.013	C	2.411	-0.342	2.546
C	3.451	1.122	-0.773	C	-3.644	1.329	-3.234	C	-2.508	1.193	-1.331

C	-2.188	-2.434	1.657	C	2.587	-4.407	-0.170	C	1.541	-0.313	1.460
C	-2.156	-3.410	-0.641	C	-4.504	1.064	-2.034	C	3.422	-0.468	-0.014
C	2.394	5.179	2.384	C	3.311	-3.690	-1.321	O	-5.164	1.926	4.464
C	1.725	-1.441	-3.401	O	-5.736	0.431	-2.407	C	-4.645	2.139	-1.943
H	7.539	0.835	-0.715	O	0.150	-3.541	5.425	H	-3.688	0.359	6.128
H	-4.206	-1.546	-0.537	H	-5.559	-0.225	0.051	C	3.793	-0.426	2.359
C	5.167	1.706	1.284	H	-0.917	3.164	-3.250	C	-4.964	2.307	-0.586
C	4.918	0.709	-1.030	H	-1.742	1.434	-4.111	C	4.292	-0.582	1.067
C	-3.627	-1.897	1.524	H	2.956	-5.951	0.974	H	5.510	-0.499	3.203
C	-3.653	-3.615	-0.319	H	4.612	0.029	-1.061	H	3.831	-0.648	-0.998
C	5.824	0.835	0.222	H	-5.663	-1.507	2.182	H	-3.667	0.424	-4.107
C	-4.310	-2.344	0.209	H	5.986	1.539	-2.412	H	2.032	-0.350	3.559
C	-4.370	0.848	-2.550	H	-4.509	-2.636	4.045	O	-6.141	2.890	-0.193
C	4.765	-2.938	3.786	H	4.918	3.176	-3.940	O	0.106	-2.731	6.102
C	-6.501	-1.505	0.619	H	1.363	-2.626	3.255	H	0.501	1.206	-6.707
H	2.685	3.265	0.707	H	0.910	5.376	-5.179	H	0.900	-2.314	3.538
H	0.032	-2.996	-2.324	H	-2.398	-3.331	5.169	H	-6.653	3.122	-0.983
H	0.459	5.134	-0.151	H	2.914	4.299	-4.743	H	5.347	-0.791	0.899
H	2.188	5.277	-0.445	H	3.179	-3.256	1.532	H	-1.879	-1.244	6.624
H	2.189	-3.967	-3.605	H	1.563	-3.950	1.667	H	3.372	-0.762	-3.768
H	0.836	-4.974	-3.163	H	4.428	-2.168	-0.256	O	4.790	0.655	-4.282
H	1.149	4.535	-2.538	H	-4.168	1.595	-4.148	C	1.241	-3.497	5.734
H	2.101	3.214	-1.903	H	1.646	-4.840	-0.543	H	-5.528	1.781	2.008
H	3.340	-5.740	-2.613	H	-4.840	2.021	-1.605	H	-3.553	2.137	-4.404
H	2.009	-6.150	-1.551	H	2.697	-3.730	-2.227	H	0.014	-1.669	1.151
H	0.839	1.068	-2.351	H	4.236	-4.236	-1.533	H	-5.359	2.453	-2.704
H	3.826	-2.696	-1.519	H	-5.503	-0.448	-2.748	C	-5.670	1.889	5.787
H	-1.797	1.175	-3.733	H	1.111	-3.523	5.296	H	-5.950	0.869	6.083
H	6.059	-3.706	0.247	H	4.032	-1.758	-1.917	H	2.674	0.131	-5.920
H	3.286	1.845	2.343					H	2.944	1.775	-5.364
H	3.398	2.210	-0.784					H	2.937	2.116	-2.865
H	-2.212	-3.475	2.001					H	3.719	0.959	-1.828
H	-2.048	-3.019	-1.657					H	2.091	-2.859	5.457
H	2.269	6.088	1.778					H	1.504	-4.082	6.618
H	2.234	5.438	3.434					H	1.020	-4.181	4.904
H	3.429	4.820	2.261					C	5.789	-0.033	-3.561
H	2.235	-0.476	-3.367					H	5.797	0.227	-2.491
H	2.227	-2.063	-4.156					H	5.676	-1.126	-3.651
H	0.683	-1.287	-3.718					H	6.751	0.259	-3.993
H	5.985	-0.160	0.662					H	-4.941	2.284	6.507
H	-3.782	-4.405	0.432					H	-6.559	2.523	5.786
H	-4.223	-2.276	2.363								
H	5.844	1.806	2.140								
H	5.331	1.370	-1.802								
H	-3.628	-0.804	1.593								
H	-4.184	-3.954	-1.216								
H	-4.036	0.606	-3.570								
H	-5.264	0.257	-2.331								
H	-4.635	1.918	-2.518								
H	3.716	-2.904	4.118								
H	5.136	-3.967	3.923								
H	5.350	-2.266	4.420								
H	-7.539	-1.849	0.661								
H	-6.263	-0.998	1.567								
H	-6.402	-0.768	-0.195								
H	4.952	-0.313	-1.421								
H	5.032	2.717	0.875								
H	-1.669	-4.390	-0.618								
H	-1.655	-1.880	2.436								
H	2.818	0.758	-1.586								
H	4.024	0.251	2.391								
H	5.394	-2.084	0.091								
H	-1.125	-0.127	-2.753								
H	5.022	-3.950	-1.859								
H	0.349	2.094	-3.682								
H	4.470	-4.385	1.789								
H	-2.569	2.358	-1.772								
H	2.585	-1.284	2.339								
H	-1.270	-0.022	1.375								
H	2.380	-3.474	2.285								
H	-2.303	1.627	0.453								
H	3.834	-5.445	-0.229								
H	-0.869	3.561	-2.075								
H	0.050	-3.459	1.432								
H	1.357	0.723	1.990								
H	0.395	-1.315	2.096								
H	0.845	-0.400	-0.751								
H	1.902	-4.981	0.823								
H	-0.989	3.360	0.120								
H	0.318	-4.538	-0.572								
H	0.176	2.414	1.716								

M9	x	y	z
C	-4.341	1.829	1.214
C	1.796	-0.274	-2.670
C	-3.342	1.489	2.283
C	-0.574	-0.565	-3.356
C	-1.491	-1.119	-4.277
C	-3.201	0.890	-2.833
O	4.921	2.072	2.448

C	-3.912	1.468	-0.183
C	-2.094	0.965	1.996
C	3.240	-0.051	-3.096
H	-5.302	1.345	1.439
C	1.398	-0.095	-1.358
C	-1.179	0.655	3.060
C	0.823	-0.600	-3.673
C	-0.426	0.267	0.305
C	-0.015	0.020	-1.049
C	-1.699	0.692	0.606
C	-1.068	-1.582	-5.510
C	-3.715	1.720	3.645
C	3.635	-1.020	-4.218
C	-1.594	0.837	4.418
C	1.223	-1.002	-4.980
C	-2.876	1.396	4.684
C	2.672	-0.888	-5.399
C	0.105	0.120	2.802
C	-0.977	-0.014	-2.075
C	0.589	-0.072	1.378
O	-2.006	-2.129	-6.344
C	-2.647	0.900	-0.465
C	2.373	0.284	-0.318
C	0.902	-0.306	3.858
H	-2.921	0.759	-3.868
C	-0.740	0.446	5.468
C	0.286	-1.483	-5.877
C	0.485	-0.139	5.195
H	-1.565	-2.444	-7.148
C	2.816	1.144	1.908
C	-2.291	0.543	-1.805
C	1.954	0.525	1.008
C	3.728	0.464	-0.647
O	-4.961	2.261	3.797
C	-4.424	1.466	-2.555
H	-3.169	1.543	5.717
C	4.127	1.444	1.526
C	-4.793	1.725	-1.228
C	4.597	1.054	0.270
H	5.792	2.230	2.053
C	4.240	-0.128	-1.935
H	-2.534	-1.244	-4.019
H	2.501	1.388	2.915
O	-6.007	2.276	-0.906
O	1.227	-0.523	6.278
H	0.604	-1.811	-6.867
H	1.860	-0.764	3.642
H	-6.514	2.408	-1.722
H	5.644	1.200	0.006
H	-1.043	0.578	6.502
H	3.595	-2.050	-3.821
O	4.974	-0.714	-4.596
C	2.503	-1.103	6.057
H	-4.581	2.902	1.262
H	4.464	-1.190	-1.741
H	0.758	-1.159	1.271
H	-5.095	1.738	-3.369
C	-5.423	2.518	5.112
H	-4.772	3.231	5.634
H	2.896	-1.634	-6.170
H	2.852	0.097	-5.855
H	3.292	0.964	-3.526
H	2.910	-1.310	7.049
H	2.431	-2.043	5.495
H	3.176	-0.417	5.527
C	5.639	-1.738	-5.307
H	6.674	-1.410	-5.439
H	5.635	-2.687	-4.748
H	5.202	-1.918	-6.300
H	-6.419	2.951	5.001
H	-5.491	1.595	5.702
H	5.186	0.329	-2.237

Supplementary Table 6. Hydrogen bonds partners with the average distance, hydrophobic interaction between ligands and WT-RBD and the variant SARS-CoV-2 virus. All the values are evaluated the last 10 ns of the MD trajectories (3 repetitions).

**Ligand *viii***						
	WT-RBD with ACE2 PDB code 7c8d	WT-RBD with nanobody PDB code 7kgj	WT-RBD with antibody PDB code 7f63	RBD Alpha variant PDB code 7neg	RBD Beta variant PDB code 7ps0	RBD Omicron variant PDB code 7wbp
**Hydrogen bonding**	GLY496(2.79Å), TYR495(2.94Å),	LEU452(2.93Å), ASN450(2.96Å), LEU452(3.00Å), SER494(3.00Å), TYR449(2.99Å)	GLN493(2.72Å), PHE456(2.90Å), GLN493(2.97Å), LEU455(2.98Å)	GLN493(2.84Å), ASN450(2.92Å), LEU452(2.95Å), TYR449(2.98Å), PHE490(2.93Å)	GLN493(2.89Å), ASN450(2.93Å), LEU452(2.91Å), PHE490(2.91Å), ASN440(2.74Å)	ARG493 (2.96Å), PHE456(2.94Å), LYS440(2.93Å)
**Hydrophobic interaction**	ARG403,TYR449, TYR495,GLY496, GLN498,TYR505, ASN501,TYR453, SER494	LEU452, THR470, PHE490, ASN450, LEU452	LEU455, PHE456, TYR489, GLN493, LEU455	TYR449, ASN450, LEU452,PHE490, GLN493,SER494, THR470	TYR449, ASN450, LEU452, LYS484, PHE490, SER494, THR470, LEU452, GLN493	LEU452, PHE490, TYR449, ARG493, SER494, LEU452, THR470, ARG493, ALA484, ASN450
**SASA protein (Å²)**	11024.8± 66.1	10978.9± 90.1	12623.8± 98.4	10733.0± 96.9	11433.1± 92.2	11367.1± 83.3
**SASA Ligand (Å²)**	870.5± 12.2	879.5± 13.9	892.5± 13.0	887.0± 12.0	892.6± 11.0	884.7± 12.6
**Radius of Gyration (Å)**	18.2 ± 0.02	18.2 ± 0.02	19.2 ± 0.02	17.6 ± 0.02	18.2 ± 0.02	18.2 ± 0.02
**Ligand *ix***						
	WT-RBD with ACE2 PDB code 7c8d	WT-RBD with nanobody PDB code 7kgj	WT-RBD with antibody PDB code 7f63	RBD Alpha variant PDB code 7neg	RBD Beta variant PDB code 7ps0	RBD Omicron variant PDB code 7wbp
**Hydrogen Bonding**	TYR449(2.93Å), SER494(2.89Å) GLU484(2.82Å), VAL483(2.99Å), GLN493(2.79Å)	LEU452(2.99Å) ASN450(2.98Å), GLU484(2.86Å)	PHE490(2.86Å), GLU484(2.78Å) GLN493(2.93Å)	LYS417(2.82Å), PHE490(2.77Å), GLN493(2.90Å)	LEU455(2.95Å), GLN493(2.96Å), PHE486(2.96Å), PHE490(2.99Å)	ALA484(2.91Å), LEU452(2.99Å)
**Hydrophobic interaction**	TYR449,ASN450, LEU452,GLU484, LEU492,PHE490, SER494, LEU490	LEU452, ILE472, GLY482, VAL483, THR470, PHE490, GLU484	PHE456, GLU484, PHE486, TYR489, PHE490, GLU484, GLN493, TYR489	TYR489, LEU455, PHE456, TYR489, GLN493, PHE490, LYS417, PHE486	LYS848, TYR489, PHE486, PHE490, PHE456, LYS484, TYR489, LEU455, GLN493	LEU452, PHE490, SER494, ILE472, ALA484, ARG493, SER494, VAL483, ARG493, GLY482
**SASA protein (Å²)**	10992.1± 72.9	10680.5± 63.3	12125.6± 85.1	10467.5± 72.0	11258.3± 73.4	11304.9± 73.6
**SASA Ligand (Å²)**	682.5± 10.6	704.6± 7.9	722.5± 7.9	718.4± 10.8	724.3± 8.4	715.5± 9.9
**Radius of Gyration (Å)**	18.2 ± 0.02	18.2 ± 0.02	19.2 ± 0.02	17.6 ± 0.02	18.2 ± 0.03	18.2 ± 0.02

Supplementary Figures

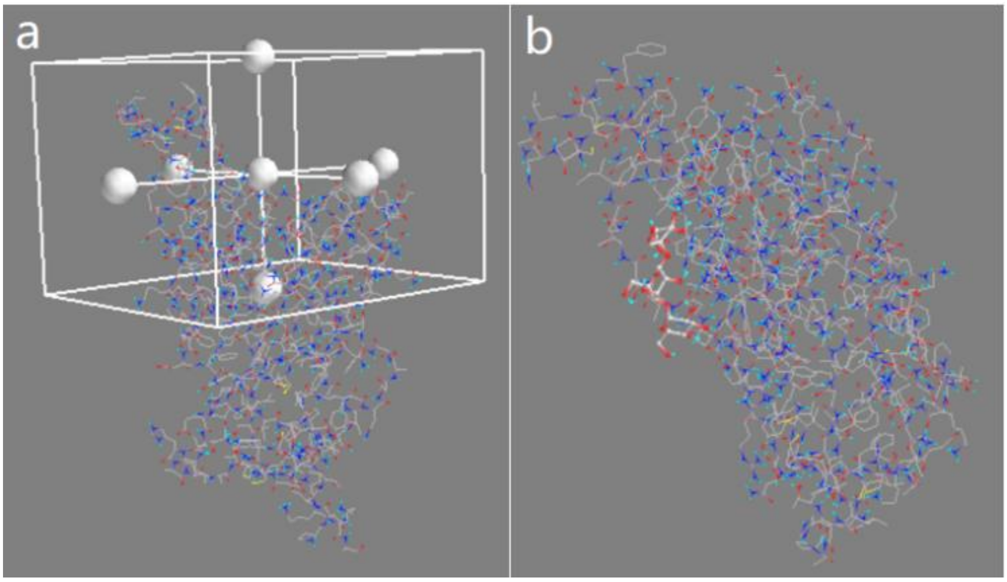

Figure S1. The vina search space defined for docking studies. (a) Top region of RBD was selected as the vina search space where is the predicted binding site of ACE2. (b) Structure of discarded ligand-RBD complex (PDB code 7F63, PubChem CID number 193471).

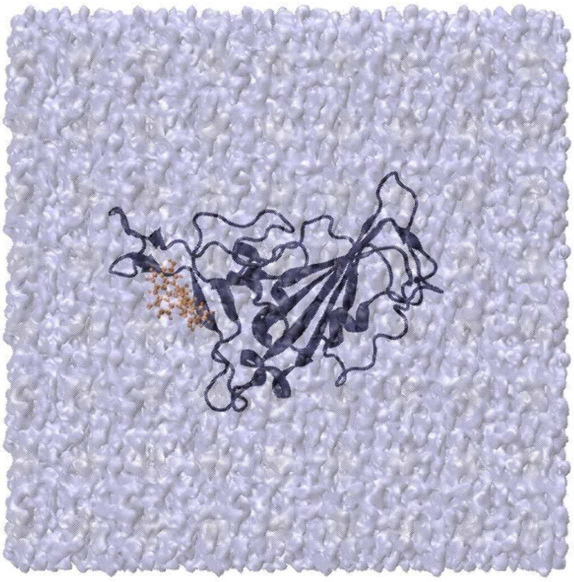

Figure S2. Visualisation of the starting model of the MD simulation of *ligand ix* / WT-RBD complex with VMD. Ligand is shown in orange, protein backbone in black and water box in iceblue.

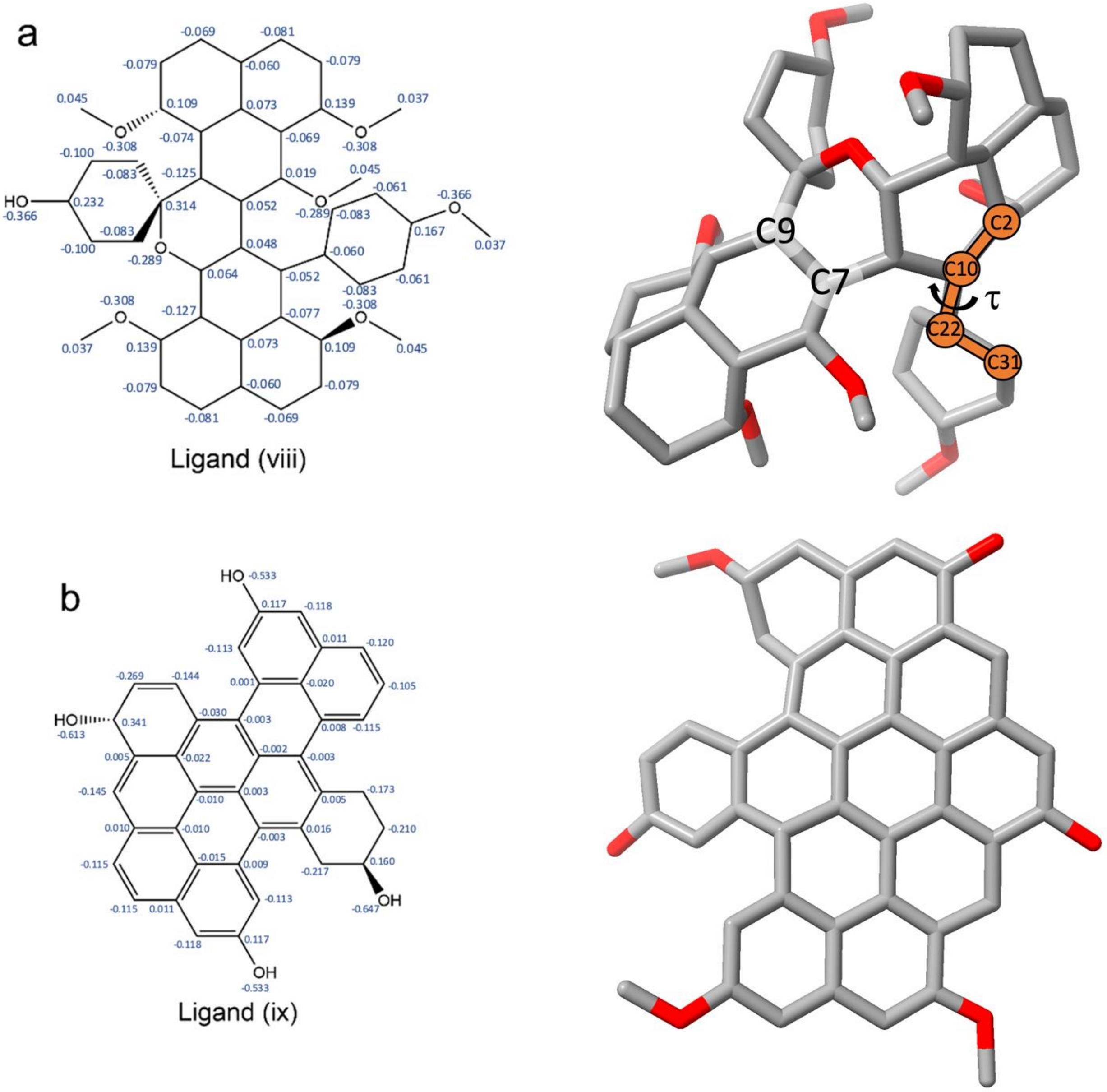

Figure S3. ESP Atomic partial charges and DFT energy minimized structures computed for a) *ligand viii* and b) *ligand ix*.Torsional angle τ used to describe the flexibility of ligand viii is highlighted in orange.

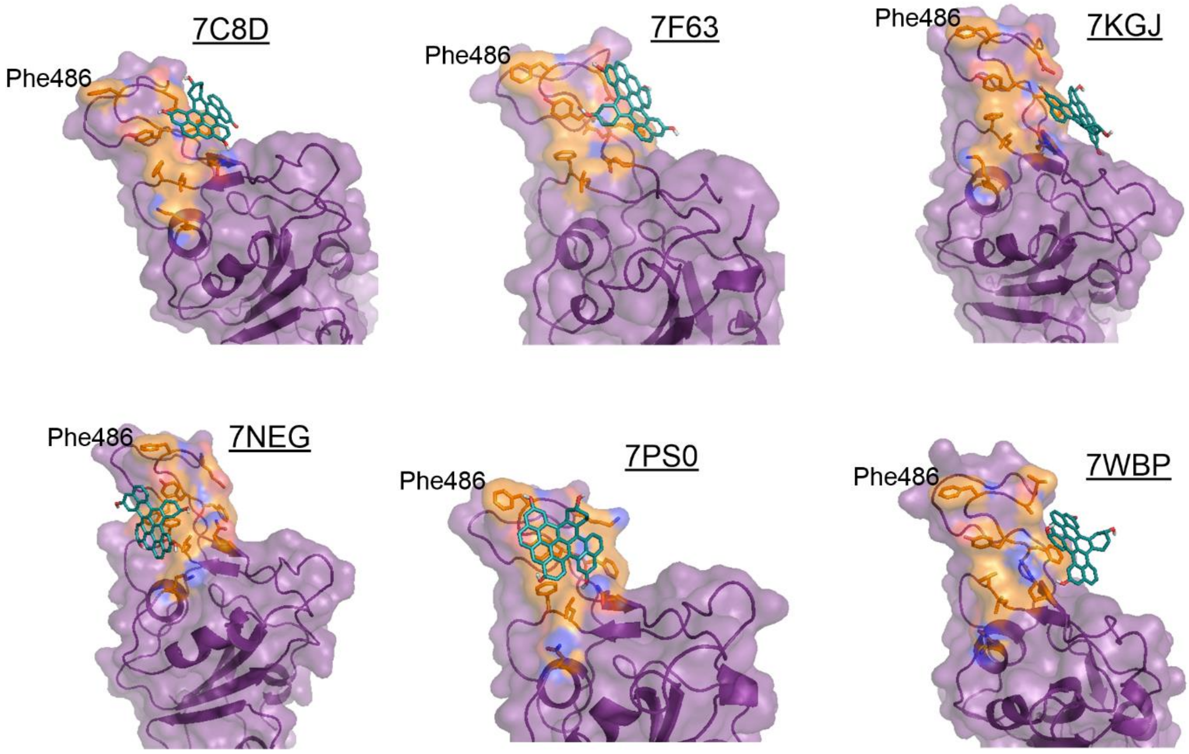

Figure S4.*Ligand ix* slide over the vulnerable pocket R1 (orange). The results of molecular docking obtain using the structures of wild-type RBD solved in the presence of ACE2 (PDB code: 78D), antibody (PDB code: 7F63) and nanobody (PDB code: 7KGJ), and the structure of Alpha (PDB Code: 7NEG), Beta (PDB Code: 7PS0) and Omicron (PDB code: 7WBP) variants.

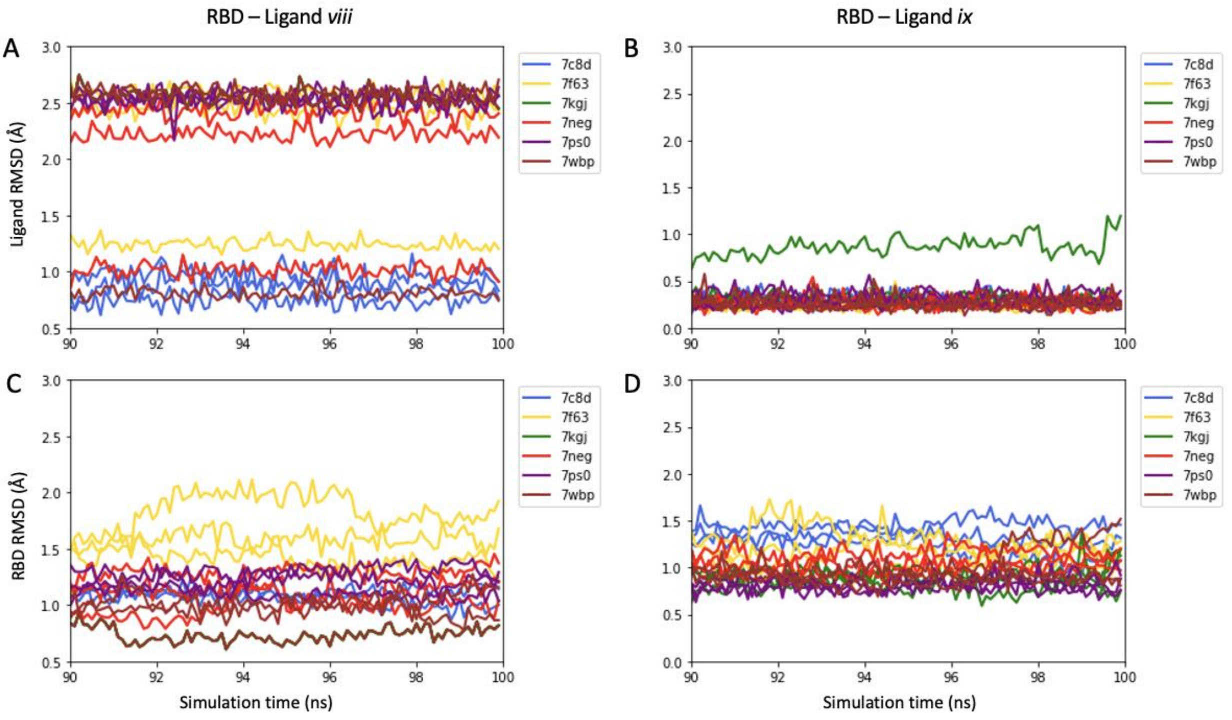

Figure S5. Evolution of root-mean-square deviation (RMSD) of the atomic positions of heavy atoms of the ligands (vii) and (ix) with respect to initial DFT optimized structures **(A,B)** and RMSD of the atomic positions of heavy atoms of the RBD docking sites in presence of ligands (viii) and (ix) with respect to the atomic coordinates of initial structural model, represented by the corresponding crystal structures: 7c8d for WT-RBD complexed to ACE2 receptor [Lili Wu, et al , *Cell Discov* **6**, 68 (2020). https://doi.org/10.1038/s41421-020-00210-9], 7f63 for WT-RBD complexed with antibody chAb-45 [Shih-Chieh Su et al Plos Pathogen 2021, https://doi.org/10.1371/journal.ppat.1009704],], 7kgj for WT-RBD complexed with nanobody Sb45 [Javeed Ahmad et al, JBC 297, p101202 ,2021, https://doi.org/10.1016/j.jbc.2021.101202], 7neg for RBD N501Y mutant of alpha variant [Supasa, P. et al, Cell 184,p2201, 2021, http://dx.doi.org/10.1016/j.cell.2021.02.033], 7ps0 RBD mutant of Beta-B.1.351 variant [Liu,C http://dx.doi.org/10.1016/j.chom.2021.11.013] and 7wbp RBD mutant of Omicron variant [Han,P. et al Cell **185**: 630-640.e10, 2022, http://dx.doi.org/10.1016/j.cell.2022.01.001] **(C,D).** RMDS were computed over the last 10 ns of MD simulations.

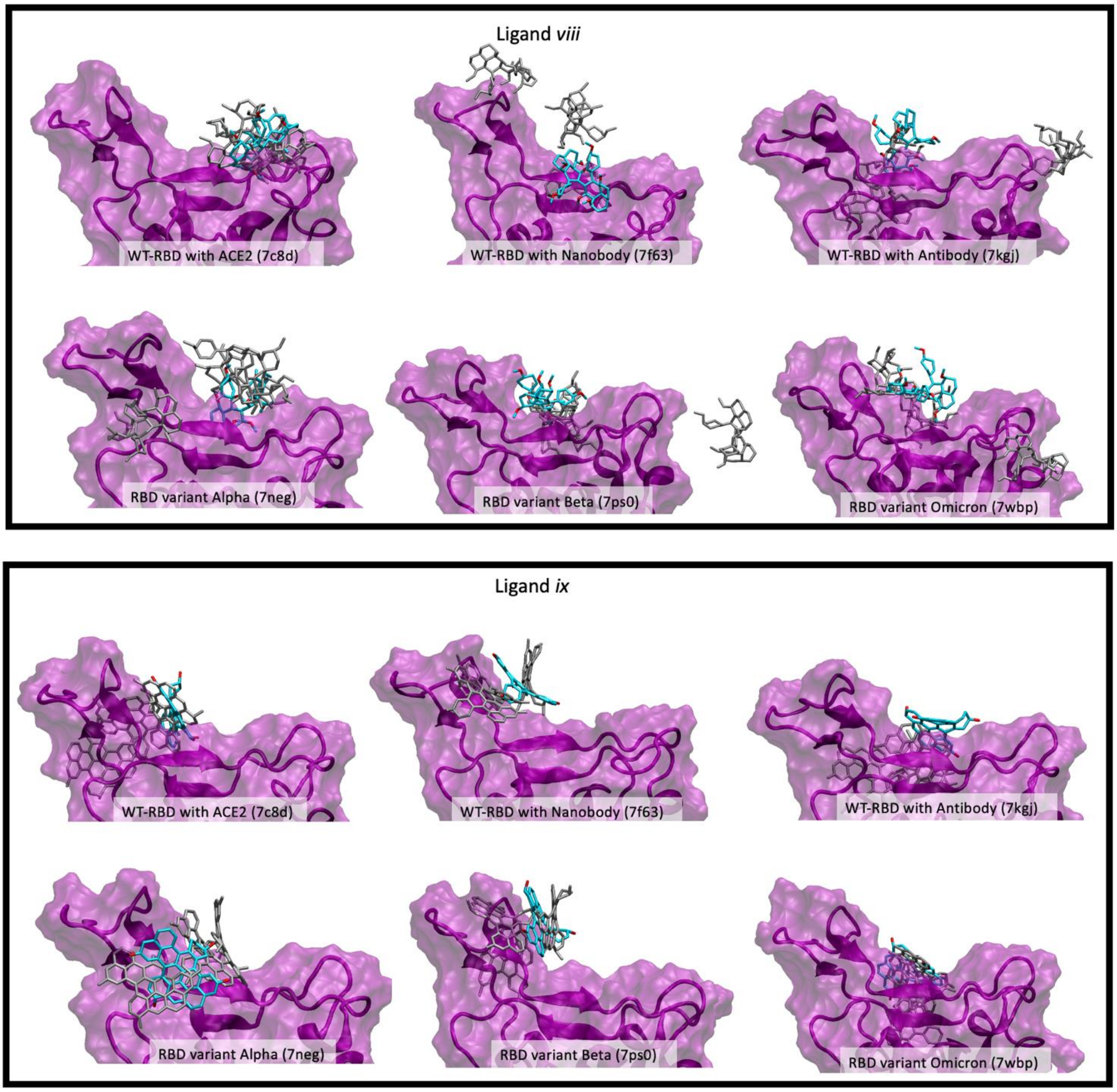

Figure S6. Conformational space of ligands viii (upper box) and ligand ix (lower box) in the six RBD model structures sampled during the repetition of MD simulations. The starting ligand-protein complex conformation is depicted with a cyan-coloured ligand while ligand-protein complexed obtained at the end of each MD-repetition is coloured gray. While flexible ligand viii adopting different conformations slides over the R2 surface and at some stages of the simulations moves away from the RBD, the rigid ligand xi interacts in almost 100% of the simulation time.

References

1. Dragelj, J., Mroginski, M. A. & Ebrahimi, K. H. Hidden in Plain Sight: Natural Products of Commensal Microbiota as an Environmental Selection Pressure for the Rise of New Variants of SARS-CoV-2. *ChemBioChem* **21**, 2946–2950 (2021).

2. Hossain, K. S. *et al.* Prospects of honey in fighting against COVID-19: pharmacological insights and therapeutic promises. *Heliyon* **6**, e05798 (2020).

3. Abedi, F. *et al.* Possible potential effects of honey and its main components against Covid-19 infection. *Dose-Response* **19**, 1559325820982423 (2021).

4. Pal, I. Dragon Fruit: The New Functional Food and Its Potential Use in Treating Co-Morbid Diseases in the COVID-19 Situation. in *Immunity Boosting Functional Foods to Combat COVID-19* 103–117 (CRC Press, 2021).

5. Gallelli, L., Zhang, L., Wang, T. & Fu, F. Severe acute lung injury related to COVID-19 infection: a review and the possible role for escin. *J. Clin. Pharmacol.* **60**, 815–825 (2020).

6. Trott, O. & Olson, A. J. AutoDock Vina: improving the speed and accuracy of docking with a new scoring function, efficient optimization, and multithreading. *J. Comput. Chem.* **31**, 455–461.

7. Dallakyan, S. & Olson, A. J. Small-molecule library screening by docking with PyRx. in *Chemical biology* 243–250 (Springer, 2015).

8. Kieseritzky, G. & Knapp, E.-W. Optimizing pKA computation in proteins with pH adapted conformations. *Proteins Struct. Funct. Bioinforma.* **71**, 1335–1348 (2008).

9. Wolf, A. *et al.* The redox-coupled proton-channel opening in cytochrome c oxidase. *Chem. Sci.* **11**, 3804–3811 (2020).

10. Vanommeslaeghe, K. *et al.* CHARMM general force field: A force field for drug-like molecules compatible with the CHARMM all-atom additive biological force fields. *J. Comput. Chem.* **31**, 671–690 (2010).

11. Jo, S., Kim, T., Iyer, V. G. & Im, W. CHARMM-GUI: a web-based graphical user interface for CHARMM. *J. Comput. Chem.* **29**, 1859–1865 (2008).

12. Dragelj, J., Mroginski, M. A. & Ebrahimi, K. H. Hidden in plain sight: Natural products of commensal microbiota as an environmental selection pressure for the rise of new variants of SARS-CoV-2. *ChemBioChem* **22**, 2946–2950 (2021).

13. Gowers, R. J. *et al.* MDAnalysis: a Python package for the rapid analysis of molecular dynamics simulations. in *Proceedings of the 15th python in science conference* vol. 98 105 (SciPy Austin, TX, 2016).

14. Humphrey, W., Dalke, A. & Schulten, K. VMD: Visual molecular dynamics. *J. Mol. Graph.* **14**, 33–38 (1996).

15. Wahyuni, D. K. *et al.* Molecular simulation of compounds from n-hexane fraction of Sonchus arvensis L. leaves as SARS-CoV-2 antiviral through inhibitor activity targeting strategic viral protein. *J Pharm Pharmacogn Res* **10**, 1126–1138 (2022).

16. Eskandari, V. Repurposing the natural compounds as potential therapeutic agents for COVID-19 based on the molecular docking study of the main protease and the receptor-binding domain of spike protein. *J. Mol. Model.* **28**, 153 (2022).

17. Pandya, M. *et al.* Unravelling Vitamin B12 as a potential inhibitor against SARS-CoV-2: A computational approach. *Informatics Med. Unlocked* **30**, 100951 (2022).

18. Dinata, R. *et al.* Repurposing immune boosting and anti-viral efficacy of Parkia bioactive entities as multi-target directed therapeutic approach for SARS-CoV-2: exploration of lead

drugs by drug likeness, molecular docking and molecular dynamics simulation methods. *J. Biomol. Struct. Dyn.* 1–39 (2023).
